# Unbiased identification of signal-activated transcription factors by barcoded synthetic tandem repeat promoter screening (BC-STAR-PROM)

**DOI:** 10.1101/gad.284828.116

**Published:** 2016-08-15

**Authors:** Pauline Gosselin, Gianpaolo Rando, Fabienne Fleury-Olela, Ueli Schibler

**Affiliations:** Department of Molecular Biology, University of Geneva, CH-1211 Geneva, Switzerland

**Keywords:** screening of synthetic tandem repeat promoters, signal-activated transcription factors, jasplakinolide, vinblastine, SRF, MRTF

## Abstract

The discovery of transcription factors (TFs) controlling pathways in health and disease is of paramount interest. We designed a widely applicable method, dubbed barcorded synthetic tandem repeat promoter screening (BC-STAR-PROM), to identify signal-activated TFs without any a priori knowledge about their properties. The BC-STAR-PROM library consists of ∼3000 luciferase expression vectors, each harboring a promoter (composed of six tandem repeats of synthetic random DNA) and an associated barcode of 20 base pairs (bp) within the 3′ untranslated mRNA region. Together, the promoter sequences encompass >400,000 bp of random DNA, a sequence complexity sufficient to capture most TFs. Cells transfected with the library are exposed to a signal, and the mRNAs that it encodes are counted by next-generation sequencing of the barcodes. This allows the simultaneous activity tracking of each of the ∼3000 synthetic promoters in a single experiment. Here we establish proof of concept for BC-STAR-PROM by applying it to the identification of TFs induced by drugs affecting actin and tubulin cytoskeleton dynamics. BC-STAR-PROM revealed that serum response factor (SRF) is the only immediate early TF induced by both actin polymerization and microtubule depolymerization. Such changes in cytoskeleton dynamics are known to occur during the cell division cycle, and real-time bioluminescence microscopy indeed revealed cell-autonomous SRF–myocardin-related TF (MRTF) activity bouts in proliferating cells.

## Abstract (precis)

Gosselin et al. designed a widely applicable method, dubbed BC-STAR-PROM, to identify signal-activated TFs without any prior knowledge of their properties. To establish proof of concept for BC-STAR-PROM, they applied it to the identification of TFs induced by drugs affecting actin and tubulin cytoskeleton dynamics.

The identification of transcription factors (TFs) responding to a specific signal is one of the first steps in dissecting the underlying regulatory networks. Ideally, this endeavor is conducted by using unbiased and systematic approaches. Both experimental and computational methods have been developed for the identification of induced TFs and their binding motifs (Geertz and Maerkl 2010), but they generally depend on already available knowledge about the TFs in question.

Recently, approaches using synthetic random DNA as promoter elements have emerged for the identification of TFs and TF-binding motifs (Reinke et al. 2008; Schlabach et al. 2010; Gerber et al. 2013). A successful and unbiased experimental strategy was developed by Gerber et al. (2013) to screen for TFs induced by blood-borne signals. This method, dubbed synthetic tandem repeat promoter screening (STAR-PROM) involves the construction of a luciferase reporter gene library with tandemly repeated synthetic promoter elements. It relies on the observation that most TF-binding sites exist at a relatively high frequency in random DNA (Reinke et al. 2008; Gerber et al. 2013). However, this strategy requires thousands of transfections of individual expression vectors and is therefore extremely time-consuming and labor-intensive. To overcome these limitations and render the search for TFs more comprehensive, we developed a barcoded version of STAR-PROM, dubbed BC-STAR-PROM, allowing the single transfection of a pool of >3000 plasmids. Our library encompasses >400,000 base pairs (bp) of synthetic random DNA, a sequence complexity that is expected to be sufficient for the identification of most TFs (Supplemental Fig. S1A,B). To establish proof of concept, we used BC-STAR-PROM for the search of TFs induced by drugs affecting the polymerization states of actin and tubulin.

Cytoskeletal dynamics affect many cellular processes (Heng and Koh 2010; Gerber et al. 2013; Akhshi et al. 2014), and agents perturbing actin or microtubule polymerization are of potential interest for chemotherapeutic treatments (Jordan and Wilson 1998). Several studies reported a link between actin or microtubule polymerization dynamics and modifications in gene expression. This regulation can be accomplished through interactions of cytoskeleton components with factors modulating the activity of specific protein kinases (Shtil et al. 1999) or TFs (Rosette and Karin 1995; Sotiropoulos et al. 1999; Rivas et al. 2004). For example, in response to serum stimulation, the GTPase RhoA is activated and promotes actin polymerization. This results in the depletion of globular actin (G-actin), which binds myocardin-related TFs (MRTF-A and MRTF-B) that serve as coactivators of serum response factor (SRF) (Posern and Treisman 2006). As a consequence, MRTFs are released into their active form, associate with chromatin-bound SRF, and stimulate the transcription of SRF target genes. In our screen for cytoskeleton-dependent TFs, we used vinblastine (Panda et al. 1996) and paclitaxel (Arnal and Wade 1995) as microtubule depolymerization and polymerization agents, respectively, and latrunculin B (Spector et al. 1989) and jasplakinolide (Holzinger 2009) as actin depolymerization and polymerization agents, respectively. While the treatment of cells with paclitaxel and latrunculin B failed to yield strongly induced TFs, BC-STAR-PROM showed that jasplakinolide and vinblastine triggered the activation of SRF, NFkB1, and FOS:JUN heterocomplexes. The activation kinetics revealed that SRF was the only immediate early TF stimulated by both actin polymerization and microtubule depolymerization. As suggested by single-cell bioluminescence recordings, such changes in cytoskeleton dynamics during the cell division cycle could also elicit cell-autonomous bursts of SRF–MRTF activation.

## Results

### Construction of the BC-STAR-PROM library

**Figure 1.**
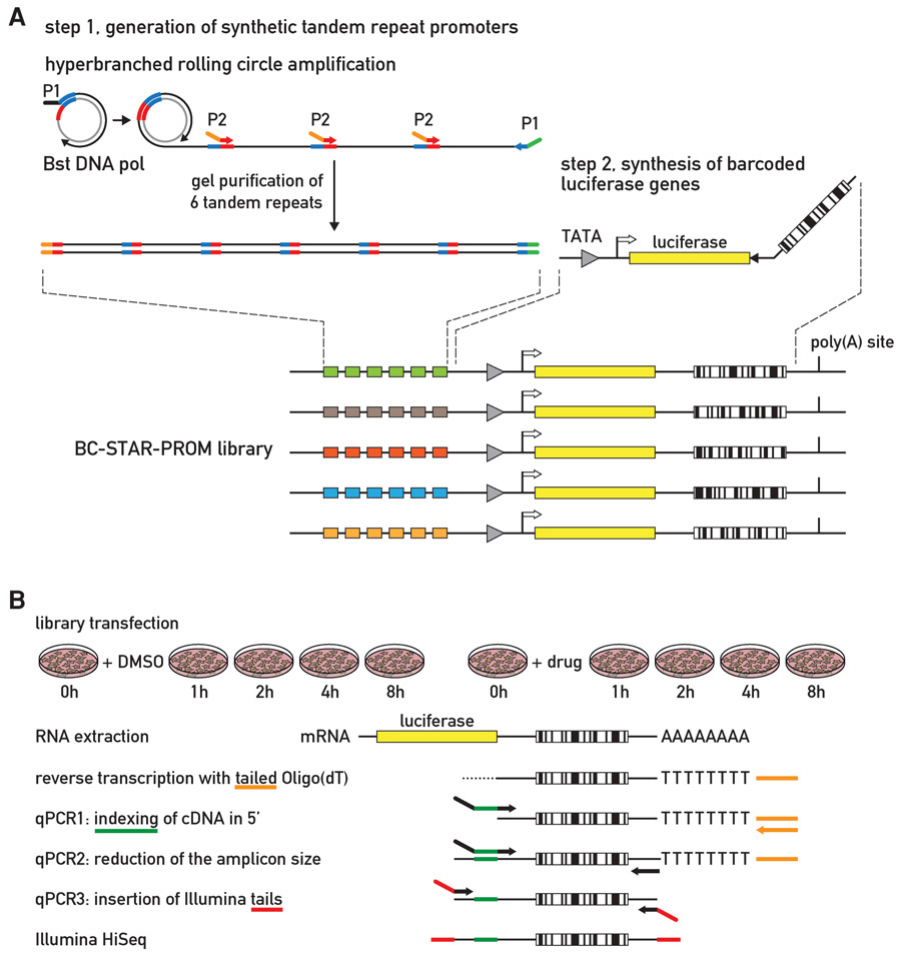
Overview of the STAR.PROM 2.0 strategy. (*A*) Construction of the BC-STAR-PROM library: Tandem repeats of 84 bp (containing 68 random base pairs) were amplified from a circular ssDNA template by hyperbranched rolling circle amplification. DNA fragments spanning six repeats were gel-purified and cloned upstream of a minimal promoter, thereby generating a STAR-PROM library of ∼4 × 10^4^ colonies. Barcoded primers (maximum complexity 3.5 × 10^9^) were used to amplify a luciferase fragment that was cloned into the STAR-PROM library. (*B*) Experimental design of TF-binding site screening: U2OS cells were transfected with the entire plasmid library containing >3000 BC-STAR-PROM plasmids. Forty-eight hours later, cells were treated with the respective drugs, and total RNA was collected at different time points. Nontreated cells (time 0) and cells treated with the vehicle (DMSO) served as controls. RNA was reverse-transcribed using a dT_15_VN-tailed primer. Using a three-step nested PCR strategy, the cDNA barcode regions were amplified using cDNA-specific primers with variable flying tails that introduced a 6-nt identifier sequence (index) for each experimental sample in the first reaction and Illumina sequencing primers in the third reaction. Barcode amplicons of an entire experiment (*n* = 10) were pooled, gel-purified, and sequenced on an Illumina HiSeq lane using the rapid mode.

We designed a potent screening strategy, dubbed BC-STAR-PROM, for the rapid and unbiased identification of signal-sensing TFs. The approach is based on (1) the high frequency of TF-binding sites in random synthetic DNA, (2) the robust activity of a given TF at promoters containing tandem repeats of its binding site, and (3) the association of the promoters’ activities with barcodes in the 3′ untranslated region (UTR) of the corresponding reporter mRNAs. We constructed a BC-STAR-PROM library containing thousands of expression vectors encompassing promoters composed of six tandem repeats of random DNA upstream of a TATA box and a luciferase ORF followed by a short barcode sequence (see Fig. 1A; Material and Methods). Two sequencing strategies were used to associate the barcodes with the corresponding promoter repeats: SMRT sequencing (Pacific Biosciences), which, owing to high read lengths (mean >6000 nucleotides [nt]), allowed the complete sequencing of a 2.3-kb restriction fragment encompassing both the barcode and the promoter repeats, and Illumina sequencing of restriction fragments, in which barcodes and promoter repeats were brought into proximity by intramolecular ligation (Material and Methods; Supplemental Fig. S2A). We thus counted 2894 promoters that were associated with 3237 barcodes (Supplemental Fig. S2B; Supplemental Table S1). We observed the associations of some promoters with multiple barcodes, but, as expected on the basis of statistical grounds, not a single barcode was linked to multiple promoters (Supplemental Table S2). The association of some promoters with multiple barcodes served as a quality control, since all barcodes linked to the same promoter yielded a similar pattern in time course experiments (Supplemental Fig. S2C).

### BC-STAR-PROM for TFs responding to cytoskeletal perturbations

To establish proof of concept for the BC-STAR-PROM approach, we searched for TFs activated by drugs engendering actin and tubulin cytoskeletal perturbations. We conducted four different studies by treating cells with drugs that induce actin stabilization (jasplakinolide), actin depolymerization (latrunculin B), microtubule stabilization (paclitaxel), and microtubule depolymerization (vinblastine). Our laboratory recently discovered that the polymerization state of the actin cytoskeleton in the liver and other tissues undergoes dramatic daily fluctuations, which in turn drive strong oscillations in the activity of MRTFs that serve as coactivators of SRF (Gerber et al. 2013). If successful, our novel BC-STAR-PROM strategy should reveal several hits for SRF–MRTF after inducing actin polymerization and perhaps additional pathways depending on other TFs. U2OS cells were transfected with the BC-STAR-PROM library, bioluminescence was recorded using a lumicycler to assess relative transfection efficiencies, and dishes with similar luciferase expression (coefficient of variation [CV] <12%) were selected for further experiments. Real-time luminescence profiles showed that these treatments were not very toxic during the 11-h recording time (Supplemental Fig. S3A). Time series (0–8 h after treatment) were established by preparing RNAs from cells treated with either the drugs or DMSO (vehicle control) (Fig. 1B). After reverse transcription of RNA, the cDNA barcode regions were PCR-amplified, indexed, and prepared for next-generation sequencing (NGS). The barcoded and indexed samples from an entire experiment (*n* = 10) were pooled, size-fractionated by agarose gel electrophoresis, and sequenced using the Illumina rapid mode. For each experimental drug, we generated 35 × 10^6^ to 135 × 10^6^ sequence reads (Supplemental Table S3), which were demultiplexed and trimmed to isolate the 20-nt barcodes (see the Material and Methods). The 3065 barcodes consistently represented in all experiments were ranked according to their relative abundance in the library and annotated starting from the most frequent barcode (Supplemental Table S1). The data produced in these experiments represent a functional screening of >400,000 bp of random DNA (3065 68mers, both DNA strands).

### Analysis of the BC-STAR-PROM results

**Figure 2.**
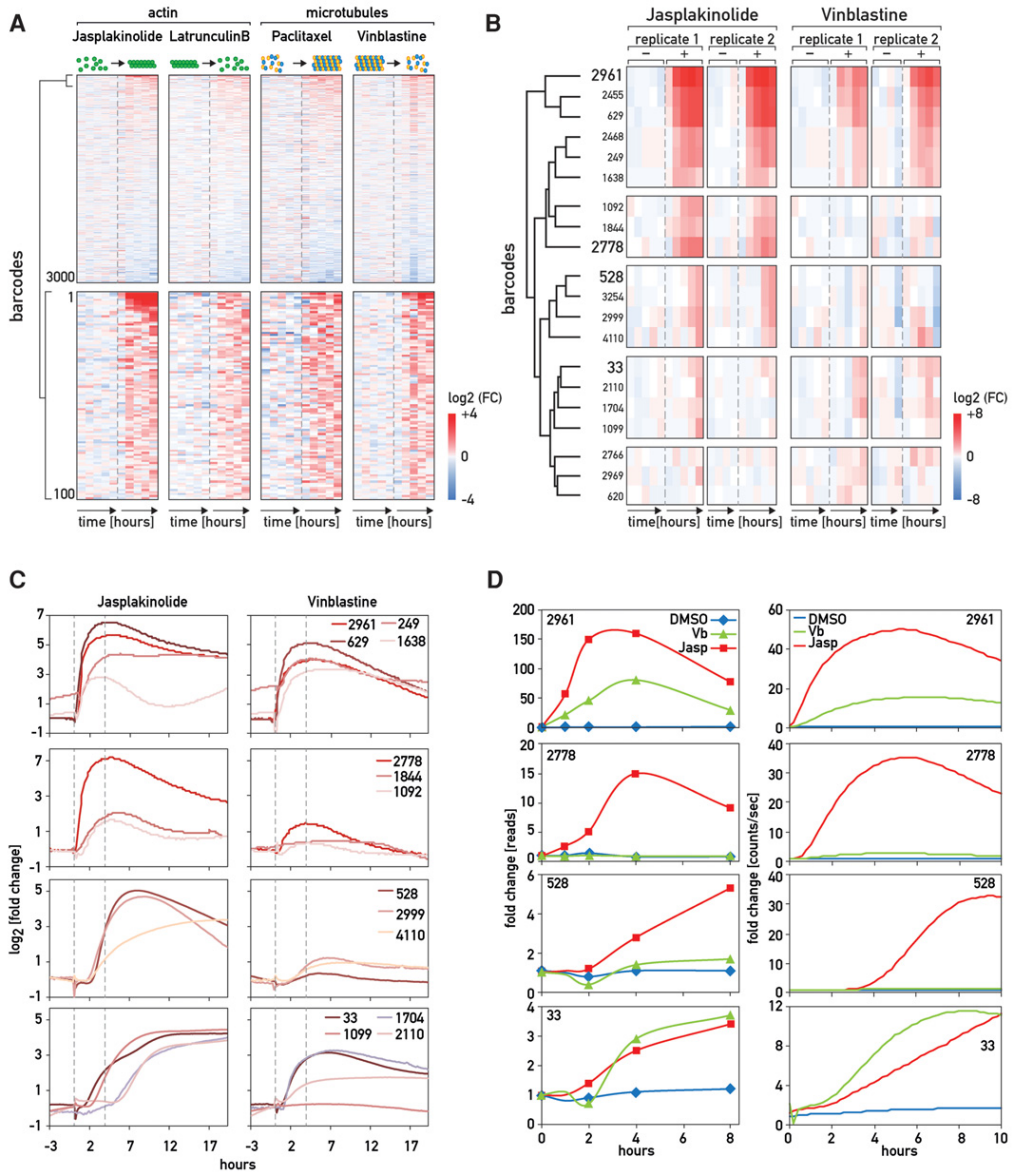
BC-STAR-PROM results obtained for drugs disturbing the cytoskeleton. (*A*) Heat map displaying the ranking of the barcode counts upon drug stimulation in time-course experiments. Each row indicates a barcode, and each column indicates the fold change calculated as the log_2_ of the barcode counts at a given time point (0, 1, 2, 4, 8 h; time specified by arrows) minus the log_2_ of the average barcode counts in the DMSO-treated controls. Fold changes were ranked according to the ratio between the mean fold change of the drug-treated samples divided by the mean fold change of the controls. The *top* panel shows the fold changes for the total library, and the *bottom* panel depicts a magnification of the 100 top-ranked barcodes. (*B*) Heat map classifying the promoters into different drug response groups. The 20 strongest promoter responses to jasplakinolide or vinblastine obtained from two biological replicates (1 and 2) were merged, and the corresponding fold changes were clustered for similarity (complete linkage and Euclidean distance). In the second biological replicate (2), DMSO time points were reduced to 0, 1, 2, and 8 h, as DMSO-treated samples did not exhibit strong fold change variation in the first replicate (1). (*C*) BC-STAR-PROM clones of interest were isolated from linearized STAR-PROM library pools. Individual STAR-PROM clones (devoid of barcodes) were transfected into U2OS cells, and their response to cytoskeletal perturbations was monitored by real-time luciferase recordings in a lumicycler. Bioluminescence curves were ordered following the clustering defined in *B* and plotted on a log_2_ scale. The first and second dotted lines indicate the times of drug treatment and maximal immediate early induction, respectively. (*D*) Barcode read counts versus photon counts. Comparison of the kinetics of drug induction recorded by barcode read counts (*left* panels) and bioluminescence (*right* panels) for four representative BC-STAR-PROM clones, one for each group of promoters shown in *B* and *C*.

Barcode counts from samples of the same experiment (i.e., with the same index) were normalized to a median value (Supplemental Fig. S3B). For each barcode, the relative expression at each time point (fold change) was calculated by dividing the read count by the average of the controls (all fold changes are in Supplemental Table S4). When ranked on their relative expression, most barcode counts were not influenced by the drug treatments, as expected (Fig. 2A, top panel; Supplemental Fig. S3A). However, the number of some barcodes was increased in the drug-treated samples, reflecting an induction of the corresponding expression vectors (Fig. 2A, bottom panel). Strongly induced BC-STAR-PROM vectors were observed in cells exposed to jasplakinolide or vinblastine but not in cells treated with latrunculin B and paclitaxel. We therefore focused on the results obtained with jasplakinolide and vinblastine. Comparison of biological replicates for these two drugs substantiated the excellent reproducibility of BC-STAR-PROM (Fig. 2B; Supplemental Fig. S3C).

To validate our results, we isolated promoters of interest from the library pool to test them individually (see the Material and Methods; Supplemental Fig. S4A). These promoters were cloned into a different vector without the barcode sequence, upstream of a basal promoter driving the expression of a destabilized luciferase (Suter et al. 2011). The resulting plasmids, carrying the 20 promoters most strongly induced by jasplakinolide and vinblastine in both biological replicates, were individually transfected into U2OS cells to examine whether the bioluminescence recordings after drug treatment faithfully reflected the barcode counts derived from BC-STAR-PROM. As shown in Figure 2B, some promoters were induced by both jasplakinolide and vinblastine. Moreover, among these plasmids, three carried a different barcode but shared the same promoter sequence and showed the same expression pattern (2455-629, 2468-249, and 528-3254). After transfection, bioluminescence was monitored in real time. Before any drug treatment, we noticed that the luciferase counts for each promoter correlated well with the corresponding number of RNA reads (corrected for the abundance of the corresponding plasmid in the library) (Supplemental Table S1) at time 0 (Supplemental Fig. S4B). Thus, promoters with low and high RNA read numbers generated low and high levels of bioluminescence, respectively. Importantly, the temporal bioluminescence patterns after drug treatment closely followed the time course of RNA reads obtained in the BC-STAR-PROM experiment (Fig. 2B–D), thereby underscoring the reliability of BC-STAR-PROM.

Promoters were categorized into four groups according to their induction kinetics and specificities of drug responses (Fig. 2B,C): promoters that responded to both jasplakinolide and vinblastine immediately and with similar kinetics (group 1: 2961-1638), promoters that were induced strongly only by jasplakinolide with an immediate early group 2: (1092-2778) or somewhat delayed (group 3: 528-4110) response, and promoters that responded to both drugs with less rapid kinetics (group 4: 1933-1704). For promoters (2766-620), BC-STAR-PROM showed a weak response when compared with the other groups; therefore, we did not analyze their individual induction profiles in the lumicycler.

### Identification and validation of drug-induced TFs

**Figure 3.**
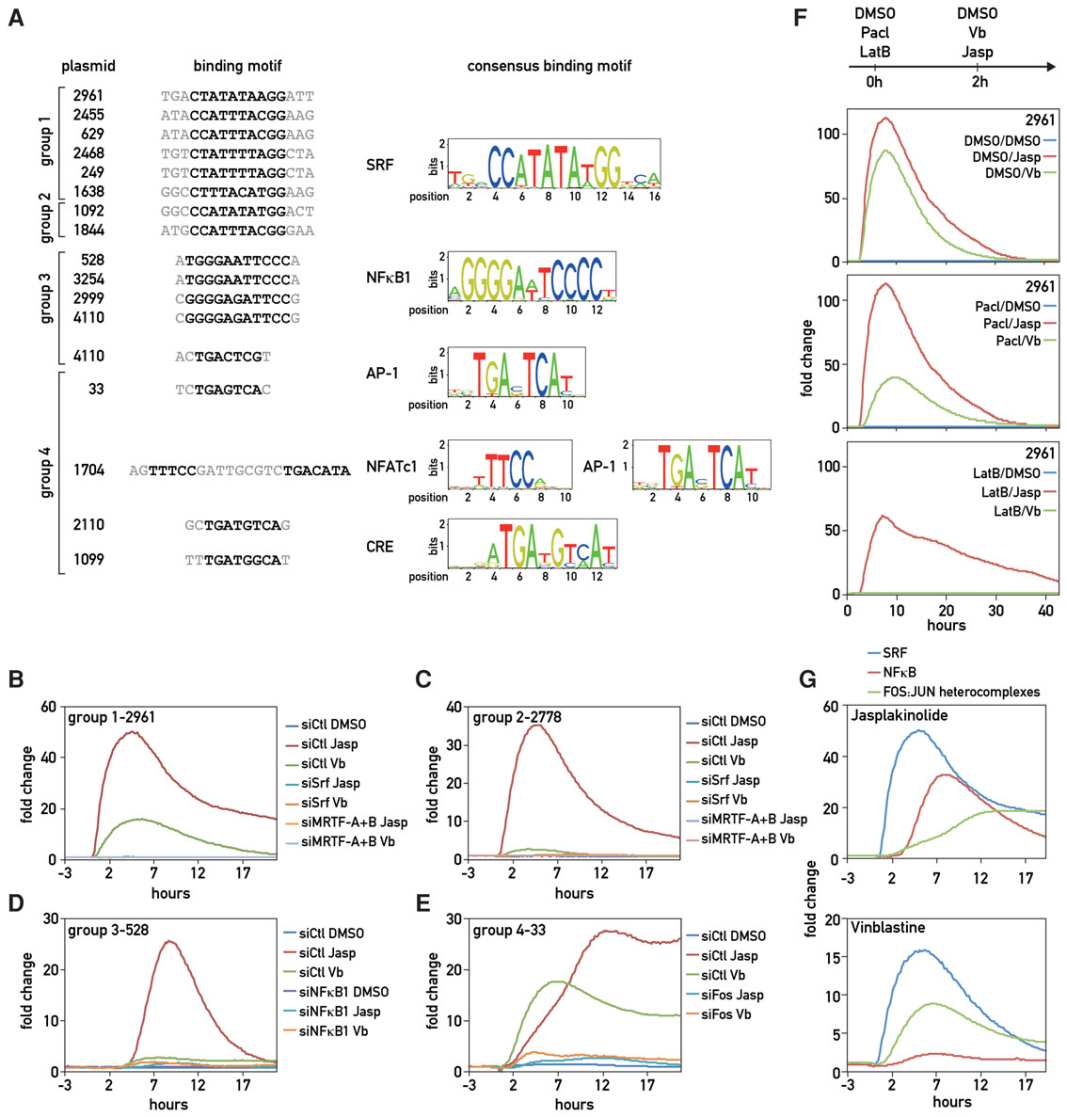
Identification of TFs responding to cytoskeletal dynamics. (*A*) Identification of shared TF-binding motifs. Sequences of promoters from the same group were examined with JASPAR for shared binding motifs. Consensus sequences (based on Weblogo, http://weblogo.berkeley.edu) are shown in the *right* panel. (*B*–*E*) Individual STAR-PROM clones were cotransfected into U2OS cells with siRNAs targeting the TFs putatively binding to the respective promoters, and the responses of the transfected cells to 0.1% DMSO, 200 nM jasplakinolide (Jasp), or 100 nM vinblastine (Vb) treatments were tested by real-time luciferase recordings. Individual real-time bioluminescence recordings are shown for one representative clone of each group of promoters. Replicates are shown in Supplemental Figure S5H. (*F*) Actin polymerization acts downstream from microtubule depolymerization in the activation of the SRF–MRTF pathway by vinblastine. U2OS cells transfected with plasmid 2961 (group 1) and grown during 2 d were pretreated with either 0.1% DMSO (control), 1 µM paclitaxel (Pacl) to stabilize microtubules, or 300 nM latrunculin B (Lat B) to destabilize actin. Two hours after the pretreatment, 100 nM vinblastine or 200 nM jasplakinolide was added, and bioluminescence was recorded during 24 h (including the 2 h of pretreatment). (*G*) Kinetics of SRF, NFkB, and FOS:JUN heterocomplex induction by jasplakinolide (*top* panel) and vinblastine (*bottom* panel).

In our library, we identified 105,791 potential matches for binding motifs, which covered 93% of the 2110 motifs described in the Transfac 2012 database. We first developed a systematic analysis of promoter sets, adapting the gene set enrichment analysis (GSEA) framework (Subramanian et al. 2005) to determine which motifs were primarily enriched at the top or bottom of the drug response rankings (Supplemental Material; Supplemental Table S5). Motifs such as SRF were significantly enriched in the induced promoter, while motifs such as CLOCK–BMAL1 were poorly ranked (Supplemental Fig. S5). However, due to the way scores were calculated, this predictive method is limited to motifs involved in an immediate early response. We therefore focused our analysis on the four groups of induced promoters, assuming that promoters showing a similar response to the drugs might share common TF-binding motifs. We scanned both strands of the promoter sequences in each group to identify such putative motifs using the JASPAR database (Mathelier et al. 2014) and then used siRNA knockdown strategies to scrutinize the validity of our TF predictions (see Supplemental Fig. S6A for knockdown efficiencies). The results for the strongest promoter of each group are presented in Figure 3, but others are shown in Supplemental Figure S6.

Promoter sequences in group 1 share an SRF-binding motif termed CArG box [CC(A/T)6GG] (Fig. 3A) and showed a strong and immediate response to both jasplakinolide and vinblastine (Fig. 2B,C). As SRF is known to respond to changes in actin dynamics via the recruitment of MRTFs released from G-actin (Sotiropoulos et al. 1999; Posern and Treisman 2006), we monitored the activity of each individual promoter in U2OS cells depleted for SRF or for MRTF-A + MRTF-B. siRNAs against both SRF and MRTFs abrogated the response of these plasmids to jasplakinolide and vinblastine (Fig. 3B; Supplemental Fig. S6B). To determine whether vinblastine induction also relied on actin dynamics, we pretreated cells for 2 h before vinblastine treatment with latrunculin B to destabilize actin filaments (Fig. 3F). As a control, cells were also pretreated with paclitaxel or DMSO and induced 2 h thereafter with jasplakinolide or vinblastine. While actin depolymerization by latrunculin B prevented a response to vinblastine, paclitaxel did not abolish the stimulatory effect of jasplakinolide. This demonstrated that SRF induction by vinblastine required the release of MRTFs from G-actin and probably relied on RhoA activation (Enomoto 1996; Chitaley and Webb 2001). Although the kinetics of the responses to the two drugs was similar, each promoter displayed a different expression profile with regard to basal level, fold change, and the duration of the activation (Fig. 2C). These differences were likely caused by sequences surrounding the CArG box, which can recruit TFs other than SRF (Fig. 3A).

The second group of promoters, which also contains an SRF-binding site, responded more vigorously to jasplakinolide than to vinblastine (Fig. 2C). For plasmids 1844 and 1092 (Fig. 2C; Supplemental Fig. S6C), the fold change of induction was already low for jasplakinolide and therefore was not visible for vinblastine (twofold) in the heat map (Fig. 2B). However, plasmid 2778 displayed a strong response to jasplakinolide (30-fold) but not to vinblastine. Both SRF depletion and MRTF-A + MRTF-B depletion attenuated the response to jasplakinolide dramatically (Fig. 3C; Supplemental Fig. S6C). Hence, SRF must have been the inducible TF for these plasmids as well. One possible explanation for the failure of plasmid 2778 to respond to vinblastine would be that its promoter sequence also contains a motif for a vinblastine-induced repressor, preventing SRF activation. To test this hypothesis, we pretreated U2OS cells with vinblastine 1 h before jasplakinolide treatment. Indeed, this pretreatment strongly dampened the jasplakinolide-mediated stimulation of plasmid 2778 expression but not that of plasmid 2961 (group 1) (Supplemental Fig. S6F). Whereas further investigations would be needed to identify this interfering factor and its mode of action, these observations suggest that BC-STAR-PROM can even reveal combinatorial effects of TF motifs.

Motif analysis for group 3 promoters revealed a shared NFkB1-binding site (Fig. 3A). These promoters displayed a strong response peaking 3 h after jasplakinolide induction (Fig. 2C) and a moderate induction in response to vinblastine. Indeed, the response to both drugs was abrogated after siRNA-mediated NFkB1 depletion (Fig. 3D; Supplemental Fig. S6D). Several studies have already shown that jasplakinolide (Kustermans et al. 2005) or vinblastine (Rosette and Karin 1995; Jackman et al. 2009) can trigger NFkB activation, but the involved mechanisms are not yet fully understood (Yu and Brown 2015). Interestingly, knocking down MRTF-A + B, but not SRF, also blunted the induction by jasplakinolide (Supplemental Fig. S6D). Hence, the induction of NFkB by jasplakinolide required MRTFs as either cofactors or transcriptional activators for NFkB, as described in Yu et al. (2014). We also showed that promoter 4110 contains binding motifs for NFkB and AP-1 and required both factors for jasplakinolide induction (Supplemental Fig. S6D). This may explain the different response kinetics of this promoter to the drug.

For the vectors of group 4, bioluminescence recording revealed a strong response 2 h after vinblastine treatment, except for plasmid 1099, which was not induced by this drug. In contrast, all examined group 4 vectors displayed a delayed response to jasplakinolide (Fig. 2C). Motif analysis detected an AP-1-binding site in promoter 33 and a CRE element in promoter 2110 and 1099, both of which serve as binding sites for FOS:JUN heterodimers (Chinenov and Kerppola 2001). Moreover, a composite NFATc1:AP-1-binding site, known to mediate synergistic binding of FOS:JUN and NFATc1 (Macian et al. 2001), was identified in promoter 1704 (Fig. 3A). Indeed, depletion of FOS in U2OS cells drastically diminished their response to both drugs but also the basal expression level of the plasmids (Fig. 3F; Supplemental Fig. S6E). Vinblastine had indeed been reported to induce AP-1, c-Jun, and ATF2 through the activation of the JNK/AP-1 pathway (Fan et al. 2001), which is also known to respond to jasplakinolide (Witteck et al. 2003). The knockdown of FOS did not affect the response of plasmid 1099 to jasplakinolide, suggesting that another TF participated in the induction process. Further investigations would be needed to identify this TF. For the plasmids affected by FOS knockdown, the inductions by both vinblastine and jasplakinolide required the presence of SRF but not MRTFs (Supplemental Fig. S6E). This is not surprising, as *c-Fos* transcription is driven by SRF–TCF rather than SRF–MRTF complexes (Zinck et al. 1993). However, both the fold change and the kinetics of the responses to jasplakinolide and vinblastine were different, indicating that these drugs elicited different mechanisms. To test whether one pathway was upstream of or downstream from the other, we pretreated cells with the antagonists latrunculin B and paclitaxel in order to destabilize the actin cytoskeleton or stabilize the microtubule network, respectively, 2 h prior vinblastine and jasplakinolide treatment (Supplemental Fig. S6G). Neither of the two pretreatments prevented the action of vinblastine and jasplakinolide on this fourth group of promoters, suggesting that no cross-talk between the actin and tubulin networks was required for the activation of TF complexes containing FOS.

Using BC-STAR-PROM, we found that most TFs were known to be induced by drugs disturbing actin and tubulin polymerization and thereby established proof of principle for the robustness of our strategy. Importantly, BC-STAR-PROM also allowed the ranking of the induced TFs in terms of kinetics and fold changes of their responses (Fig. 3G) and provided insights into the interplay between tubulin and actin cytoskeleton dynamics.

### The SRF–MRTF pathway is endogenously activated during the cell cycle

Cytoskeletal drug studies with BC-STAR-PROM emphasized that SRF is a central regulator in the immediate early response to actin polymerization and tubulin depolymerization. Intriguingly, such changes in cytoskeleton dynamics also occur during cell cycle progression (Heng and Koh 2010; Bendris et al. 2015). To examine whether intrinsically controlled cytoskeletal rearrangements might trigger endogenous SRF activity, we investigated the possible stimulation of the SRF–MRTF pathway during the cell division cycle by time-lapse microscopy.

**Figure 4.**
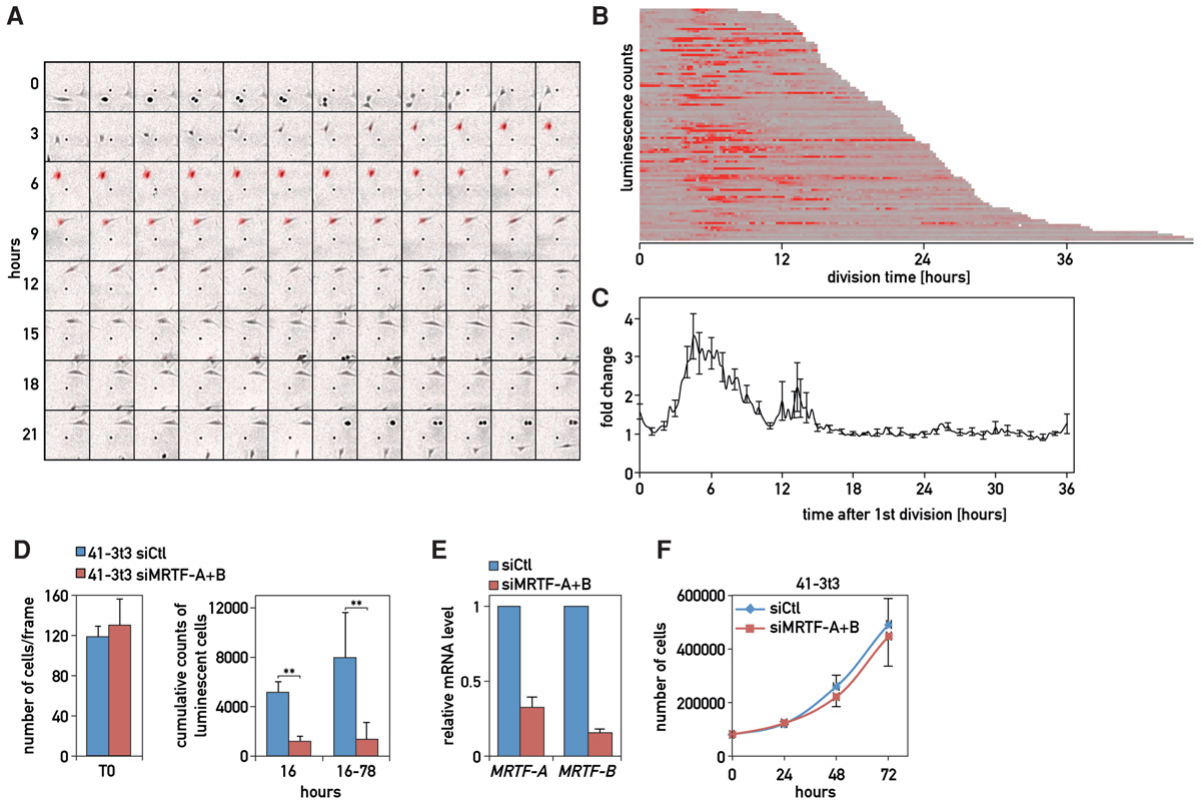
Endogenous activation of the SRF–MRTF pathway during cell cycle progression. For every time-lapse microscopy experiment, 100,000 cells were seeded in 3.5-cm dishes with white DMEM containing 20% FBS, and recording was initiated 1 h after seeding. Frames were taken at 15-min intervals. (*A*) Real-time bioluminescence (shown in red) imaging of an individual 41-3T3 cell between two cell divisions (cytokinesis) (Supplemental Movie 3). (*B*) Bioluminescence recordings for 100 cells (obtained from five different movies) were plotted from one cell division cycle (cytokinesis to cytokinesis). The luciferase signals are depicted in red. (*C*) Average curve of bioluminescence counts for the traces shown in *B* against time after the first cell division. Bioluminescence traces were recorded 16 h after plating the cells to avoid the serum effect from the medium change. Error bars represent ±SEM. (*D*) Quantification of luminescent cells per frame on time-lapse microscopy of 41-3T3 cells 48 h after transfection with siCtl or siMRTF-A + B siRNAs. The first frame was used to estimate the starting number of cells in the movies (T0). The histograms show the cumulative counts of luminescent cells measured in movies for the first 64 frames (16 h of serum response) and the period from frame 65 to 310 (16–78 h of cell cycle-dependent SRF activation). SEMs were calculated from results obtained in three different movies for each condition. (*E*) MRTF knockdown efficiencies calculated for three independent transfection experiments. (*F*) In parallel, cells were grown as in *B* and counted using a Mallassez chamber. Total cell number was plotted versus time. Error bars indicate ±SD. *n* = 3.

We first performed real-time luminescence recordings with U2OS cells transfected transiently with plasmid 2961—responding to jasplakinolide and vinblastine via the activation of SRF–MRTF pathway (Fig. 3B)—or a control plasmid (309) that does not respond to either of these drugs (Supplemental Fig. S7A,B). Cells were grown at low density in 20% FBS (to foster proliferation) and recorded in an LV200 bioluminescence microscope. While cells transfected with the control plasmid displayed a rather smooth luciferase expression profile, plasmid 2961 showed a burst of activation in between two cytokinesis events (Supplemental Fig. S7C,D; Supplemental Movies 1, 2). This indicated that promoter 2961, which contained SRF-binding motifs, was stimulated by cell-autonomous mechanisms during the cell division cycle. To record a larger number of cells, we used a stable NIH3T3 cell line (dubbed 41-3t3) expressing a single copy of a luciferase transgene driven by a synthetic promoter harboring eight 84-bp tandem repeats with CArG boxes (Gerber et al. 2013). In this transgene, the normal firefly luciferase was replaced by a destabilized luciferase (Suter et al. 2011), affording a high temporal resolution. Individual cells were monitored by real-time bioluminescence imaging during 78 h. As indicated by its name, SRF is induced by serum, and we thus had to eliminate serum-confounding effects triggered by exposing the cells to new medium (Supplemental Fig. S7E). Therefore, we evaluated only recordings obtained 16 h after plating, when the initial response to the medium change had vanished. As shown in Figure 4A, we noticed a recurrent induction of the SRF-luc reporter gene after cell division (Supplemental Movie 3). The number of cells displaying the burst of SRF activity decreased strikingly when cells reached confluency (see Supplemental Movie 4). The bioluminescence traces of >100 individual cells between two cell divisions revealed a major peak of bioluminescence centered at 5–7 h after cytokinesis in a high fraction of cells, and the timing of this SRF activation burst did not depend on the duration of the cell division cycle (Fig. 4B,C; Supplemental Fig. S7F). In a small minority of cells, a less pronounced peak centered at ∼13 h after cytokinesis was also observed (Fig. 4B; Supplemental Fig. S7F). 41-3T3 transiently transfected with siRNAs against MRTF-A + B showed almost no response to serum induction during the first 16 h (Fig. 4D; Supplemental Fig. S7E), and the cumulative number of cells expressing luciferase in dividing cells per frame for 15 min was reduced by eightfold (Fig. 4D, right panel; Supplemental Movies 5, 6). Hence, the cell cycle-dependent activation of the SRF-dependent promoter required the MRTF pathway. Cell counting performed in parallel revealed that the MRTF-A + B double knockdown had only a modest anti-proliferative effect (Fig. 4E). We therefore concluded that, during cell cycle progression, endogenous events triggered the release of MRTFs from G-actin, which led to the transcriptional stimulation of SRF target genes. In a few cells, SRF induction preceded cytokinesis (Fig. 4B), raising the possibility that the activity of SRF might also be induced just before cytokinesis, when actin forms the contractile ring.

## Discussion

### BC-STAR-PROM as the method of choice for the identification of signal-activated TFs

In this study, we showed that BC-STAR-PROM allows the identification of *cis*-acting elements for TFs responding to any stimulus of interest. In contrast to the original STAR-PROM procedure, which requires thousands of individual transfections, BC-STAR-PROM involves only a small number of transfections. In addition, it samples ∼400,000 bp of synthetic random DNA sequences rather than the 57,000 bp covered by the original STAR-PROM method. Therefore, it should reveal even TFs binding to complex DNA motifs. In this report, we established proof of principle for BC-STAR-PROM by studying its response to drugs affecting the dynamics of cellular actin and tubulin networks. Biological replicates with the cytoskeletal drugs jasplakinolide and vinblastine confirmed its excellent reproducibility (Fig. 2B; Supplemental Fig. S2C). When focusing on the plasmids strongly induced by vinblastine and jasplakinolide, we were able to identify most of the TFs known to be induced by these two drugs. A major virtue of our strategy is that it affords the monitoring of the TF induction kinetics after drug treatment (Fig. 3G), which provides information on the involved mechanism. A very rapid induction is likely to reflect an immediate early response, depending on a TF already present in an inactive form in the noninduced state, while a delayed induction favors a scenario in which the TF of interest has to be synthesized before it can elicit the response. It is perhaps surprising that polymerizing and depolymerizing the actin and tubulin cytoskeletons did not affect the activity of more TFs than the ones revealed by BC-STAR-PROM. However, as multiple hits were scored for all of the identified TFs, we feel confident that our screen approached saturation.

While we searched here for TFs whose activities were induced after the treatment of cells with drugs perturbing the actin and tubulin cytoskeletons, the screen could be adapted to the identification of any TF responding to a signal of interest. Our study focused only on TFs whose transactivation potential was increased by the drug treatment. The screening for signal-dependent repressors is technically more challenging. Obviously, in such experiments, the time-dependent fold changes depend on the mRNA half-life, and even a complete repression would reduce the barcode read number by only a factor of two for each time interval equivalent to the mRNA half-life. Hence, for the search of repressors, BC-STAR-PROM plasmids encoding extremely short-lived reporter mRNAs should be designed.

In our screen, we were able to identify the relevant TFs by a candidate approach based on the sequence of the TF-binding sites. In other situations, however, the responding promoter sequences may not be attributable to known TF-binding motifs. In such cases, the promoter repeats could serve as capture probes for DNA affinity purification, and the bound proteins could then be identified by mass spectrometry (Tacheny et al. 2012).

### BC-STAR-PROM did not reveal YAP as an actin polymerization-sensitive factor

A previous study reported that, in HeLa cells, YAP, a coactivator of TEAD in the Hippo signaling pathway, is activated by jasplakinolide (Reddy et al. 2013). However, our BC-STAR-PROM performed in U2OS did not reveal TEAD-binding sites as *cis*-acting elements conferring a potent induction by jasplakinolide. Although sequences perfectly matching the TEAD consensus binding sequence were present in many promoters of the BC-STAR-PROM library, these motifs were not enriched in jasplakinolide-responsive promoters. Putative TEAD-binding sites were found in addition to CArG boxes and NFkB1-binding sites in the promoters of plasmids 2961, 2999, and 528, whose transcription was induced by jasplakinolide (Supplemental Fig. S8). However, in contrast to SRF and NFkB1 depletion, which abrogated the induction by jasplakinolide, the depletion of YAP had little if any effect on this process (Supplemental Fig. S8B,C). We determined the expression of the endogenous YAP target genes *CNN1* and *CNN2* to examine whether the Hippo pathway was activated in U2OS cells under the conditions used for BC-STAR-PROM. As depicted in Supplemental Figure S8D, the accumulation of mRNAs encoded by these two genes was increased by merely twofold for *CNN1* and threefold for *CNN2.* Moreover, *CNN1* and *CNN2* mRNA accumulation could be detected only during the first 2 h after drug treatment. Considering the modest response of the Hippo pathway to jasplakinolide and the bioinformatics algorithms used for the BC-STAR-PROM data mining, it is not surprising that YAP was not detected in our screen.

### Endogenous activation of the MRTF–SRF pathway during the cell cycle

SRF was the only TF responding to both polymerization of actin and depolymerization of microtubules through the same pathway. Cross-talk between actin and tubulin dynamics is known to occur during the cell division cycle (Akhshi et al. 2014). In fact, microtubule depolymerization triggers RhoA activation (Chitaley and Webb 2001), and Rho GTPases have been reported to regulate cell cycle progression at two stages: one at the G1/S transition and the other during cytokinesis (Heng and Koh 2010). Our real-time bioluminescence recordings of single cells revealed that SRF-dependent promoters were indeed induced via the MRTF pathway in a large fraction of dividing cells ∼6 h after cytokinesis, which, in NIH3T3 cells, coincides roughly with the G1/S transition. The integrity of the actin cytoskeleton and RhoA activation are required for the G1/S transition (Heng and Koh 2010), but whether microtubules regulate the activation of RhoA and actin polymerization during G1/S transition is less clear. Nonetheless, the depolymerization of microtubules induces the entry of quiescent cells into S phase, perhaps by provoking actin polymerization (Bershadsky et al. 1996), and several MRTF/SRF target genes encode regulators of cytoskeleton dynamics and the cell cycle (Esnault et al. 2014). Indeed, MRTFs have been demonstrated to be required for the precise timing of cell cycle phases (Shaposhnikov et al. 2013). As activation of the SRF/MRTF pathway can efficiently reset circadian clocks in mammalian cells (Gerber et al. 2013; Esnault et al. 2014), it is enticing to speculate that this signaling pathway also participates in the coupling of circadian and cell cycle oscillators (Bieler et al. 2014).

We demonstrated here that BC-STAR-PROM is a powerful and unbiased methodology to identify signal-responsive TFs. Given its simple technical requirements and its independence on any a priori knowledge about the TFs of interest, BC-STAR-PROM can serve as a method of choice in the investigation of cellular signaling pathways in a large variety of biological systems.

## Materials and methods

### Cell lines, transfections, RNAi, and drug treatments

U2OS cells and the 41-3T3 cell line (Gerber et al. 2013) were maintained in DMEM supplemented with 10% FBS (Gibco 10270), antibiotics (10,000 U/mL penicillin, 10,000 µg/mL streptomycin), and 29.2 µg/mL glutamine. For the testing of individual promoters, U2OS cells grown in 3.5-cm dishes were cotransfected with 500 ng of plasmid and 60 pmol of siGENOME SMART-Pool siRNAs (Dharmacon) targeting human *MRTF-A*, *MRTF-B*, *NFkB1*, or *C-FOS* mRNA or with siGENOME nontargeting control siRNAs (Ctl) using Lipofectamine 2000 (Invitrogen). Twenty-four hours after transfection, the cell culture medium was replaced with serum-free medium, and bioluminescence was recorded in real time during a 24-h starvation period and a 24-h period after drug stimulation (1:1000 DMSO, 200 nM jasplakinolide or 100 nM vinblastine). The knockdown experiments in the 41-3T3 cell line were performed using 60 pmol of siRNA targeting SRF (siGENOME SMART pool Dharmacon), MRTF-A (GGGCUCUGCCCAUGCUUUU) (Sigma Aldrich), MRTF-B (GAAGAGCCUUCUCCAAUUU) (Sigma Aldrich), or siRNA nontargeting control (TTCTCCGAACGTGTCACGT) using Lipofectamine RNAimax (Invitrogen) according to the manufacturer's instructions. All knockdown efficiencies were assessed by RT-qPCR using SYBR Green (Invitrogen). Relative cDNA abundances were calculated by the comparative Ct method (ΔΔCt) (Livak and Schmittgen 2001) using *GAPDH* cDNA levels for normalization. The sequences of qPCR primers are in Supplemental Table S6.

### BC-STAR-PROM construction

#### pMC-Luc vector

A 2.5-kb minP-Luciferase2-SV40-poly(A) fragment (EcoRV–BamHI fragment from pGL4.23 [Promega]) was inserted between the SwaI and BamHI sites of plasmid pMC.BESPX-MCS2 (System Biosciences). In a second step, a 238-bp α-fetoprotein MERII enhancer region (Wooddell et al. 2008), produced by PCR from mouse genomic DNA (chromosome 5: 90485715-934) using the primers 5′-TCTACTAGTCATCTTTTTGATGGCAGAGTTCAGTTTACCGGGTC-3′ and 5′-TCTACTAGTGCCCACAAGAAGAGATAAAGTGGAGCTTTG-3′, was inserted into the SpeI site of the above expression vector. While this liver-specific enhancer probably had no impact in our experiments, it is likely to facilitate BC-STAR-PROM screens in livers of mice in which the library had been delivered to hepatocytes by hydrodynamic injection (Wooddell et al. 2008). To generate single HindIII and XbaI sites in the plasmid for further cloning, two consecutive site-directed mutagenesis assays were used to eliminate an extra HindIII site within the MERII region (5′-GAGCAATGGCACAGCAAC*G*TTTAACCCTGCAG-3′) and an extra XbaI site (5′-CAGATCTGATATCTCTG*GAGTCGAGCTAGCTTC-3′) in the multicloning site. Mutagenesis was verified by DNA sequencing and restriction mapping.

#### pMC-STAR-PROM-Luc library

A 524-bp insert was cloned into the dephosphorylated NheI and HindIII sites of the pMC-Luc vector. The inserts, spanning 6× tandem repeats of random 68mers flanked by 8 bp of defined sequences on both ends, were obtained by hyperbranched rolling circle amplification of ssDNA circles, as described in Wang et al. (2005) and Gerber et al. (2013). Fifty nanograms of ligation product was transformed in 4 × 50 µL of highly competent bacteria (5A, Zymo Research), resulting in ∼40,000 colonies of similar size. All STAR-PROM colonies were pooled with a cell scraper, and ∼200 µg of plasmid DNA was extracted by standard alkaline lysis and purified by spermidine precipitation, as described in Gerber et al. (2013).

#### BC-pMC-STAR-PROM-Luc library

The BC-pMC-STAR-PROM-Luc library was obtained by insertion of a barcoded luciferase insert (HindIII–XbaI fragment from pMC-Luc vector) between the HindIII and XbaI sites of the pMC-STAR-PROM-Luc library. The barcoded inserts were amplified by PCR from the pMC-Luc vector using the primer 5′-GACGTGAATTCGAGCTAGCTTCGAAT-3′ and the barcode-containing primer 5′-GGCAGTCTAGAHHDDDDHHBBVVDDDDHDBVATTATTACACGGCGATCTTGCCG-3′. In the 20-nt barcode region, only 3 nt (H, D, V, and B) were allowed at each position. This strategy prevented the formation of long homopolymeric stretches and facilitated the identification of sequencing errors at later stages. To minimize the number of promoter plasmids associated with the same barcode inserts, the number of possible barcodes was in large excess over the number of plasmids. As a consequence, some promoters could be linked to more than one barcode, which may serve as a useful control in verifying the correctness of the bioinformatics pipeline. Cloning was conducted by transforming 6 × 100 µL of ZYCY10P3S2T competent cells (Thermo Fischer Scientific), resulting in ∼5000 colonies distributed on six plates. Plasmid DNA was prepared from separate subcultures of ∼2300 and ∼2700 colonies. Bacterial vials were prepared from these two subcultures to ensure library conservation and storage.

### Association of promoters with barcodes

After digestion of 20 µg of the library DNA, a 2275-bp NheI–XbaI fragment was gel-purified on a 1% agarose gel using spin columns (QIAquick gel extraction kit, Qiagen) to extract DNA from agarose. The NheI and XbaI sites mark the beginning of the promoter and the end of the barcode, respectively. Two sequencing methods were used to assign barcodes to promoter sequences. We first performed a “long read” sequencing strategy (SMRT, Pacific Biosciences, Genomic Technologies Facility of the University of Lausanne), in which the initial NheI–XbaI fragments of 2.3 kb were ligated to hairpin adapters. We obtained 40,000 reads that confirmed the 6× tandem repeat nature of our promoters and established the barcode–promoter associations.

In a second strategy, 400 ng of NheI–XbaI DNA fragments was ligated for 90 min at room temperature at a low concentration (1 ng of DNA per microliter) to favor intramolecular ligation, thereby forming circles with barcodes in close proximity to promoters. The treatment of the ligation products with exonucleases (5 U of ExoVII, 100 U of ExoIII; Affymetrix) for 90 min at 37°C removed linear DNA molecules. Enzymes were inactivated by adding 1 µL of 0.5 M EDTA (pH 8.0) and 1 µL of SDS (20%), DNA circles were extracted with phenol/chloroform and precipitated overnight at −20°C with 2.5 vol of ethanol in the presence of 0.3 M NaCl. Precipitated circles were washed with 75% ethanol, resuspended in 30 µL of 10 mM Tris/HCl (pH 8.0), and divided into three aliquots that were independently digested with ApaI, FspI, or HincII for 150 min at 37°C to prepare linear templates for PCR reactions. The three digestions were heat-inactivated for 15 min at 70°C and pooled. The independent digestion with three different restriction endonucleases reduced the possibility of cleaving the DNA regions encompassing the promoter and/or barcode regions. DNA fragments spanning the barcode/promoter junction were PCR-amplified from 10 ng of linearized templates using Vent polymerase (New England Biolabs) and primers 37 forward and 72 reverse. The PCR products were diluted 1:100 in 10 mM Tris (pH 8), and Illumina sequence tails were introduced by a second PCR with primers 44 forward and 45 reverse. All primer sequences are in Supplemental Table S6. The final DNA amplicons were gel-purified on a 1.5% agarose gel using spin columns (Qiagen), concentrated by ethanol precipitation in the presence of 0.2 M NaCl, and quantified by UV absorbance at 260 nm. Two-hundred nanograms of DNA was sent for sequencing to the Genomics Platform of the University of Geneva. The library was loaded on a MiSeq flow cell to produce 5 million reads of 150 bp, each covering the barcode and the first promoter repeat. Barcodes and their corresponding promoters identified by both methods, displaying >50 reads in MiSeq data (Supplemental Fig. S2B), are in Supplemental Table S1.

### BC-STAR-PROM

U2OS cells were transfected with 2 µg of the BC-STAR-PROM library using 5 µL of Xtremegene 9.0 (Roche). Twelve hours after transfection, cells were serum-starved for 24 h. Relative transfection efficiencies were monitored by measuring bioluminescence using a lumicycler (ActiMetrics), and dishes producing photon counts whose CV was within 12% were treated with drugs or the vehicle DMSO. The final drug concentrations were 100 nM vinblastine, 100 nM paclitaxel, 300 nM latrunculin B, and 200 nM jasplakinolide. The final DMSO concentration was 1:1000. At times 0, 1, 2, 4, and 8 h after the treatment, cells were collected in 1 mL of Trizol (Invitrogen) and stored at −80°C until RNA extraction.

### RNA preparation

RNA was extracted with TRIzol (Life Technologies) following the manufacturer's protocol. To reduce the contamination of plasmid DNA in RNA preparations (Botvinnik et al. 2010), the RNA was dissolved and precipitated in 2.5 M LiCl overnight at −20°C. RNA (2 µg) was reverse-transcribed using SuperScript II (Invitrogen) and an oligo(dT) tailed primer (5′-ACGGCTAGCACGTTTTTTTTTTTTTTTVN-3′, where V is C, G, or A). This resulted in cDNAs of mRNAs starting at the polyadenylation cleavage site.

### Illumina sequencing library preparation

To maximize the coverage of RNA barcodes, the cDNA was amplified in eight 250-ng RT-qPCR reactions. cDNA amplification was carried out in a qPCR machine (LightCycler 480, Roche), monitored in real time with SYBR Green chemistry, and stopped in the exponential phase of amplification to minimize the number of amplification cycles. The forward primers had a 6-nt index tail specific for every sample (i.e., time point, drug, or DMSO) to recognize the origin of the sample in multiplexed sequencing reactions. All indexes had a Hamming distance of 3, a measure adopted from signal processing to indicate that at least three sequencing errors are required to convert an index into another one. As revealed in control PCR reactions with RNA that was not reverse-transcribed, contaminating plasmid DNA was not amplified, as the first PCR reactions were carried out using the oligo(dT)-containing primer. A second round of qPCR reduced the amplicon size to 99 bp. At this point, amplicons of samples from a single experiment were pooled together and used as templates for a third PCR reaction that introduced the Illumina sequencing tails. All primer sequences are in Supplemental Table S6. The final amplicons were gel-purified on a 2% agarose gel (using Qiagen spin columns), concentrated by ethanol precipitation in the presence of 0.250 M sodium acetate (pH 5.2), and quantified by UV absorbance at 260 nm. This DNA (∼200 ng) was sent for sequencing to the Genomics Platform of the University of Geneva.

### NGS

Before sequencing sizes were confirmed with a bioanalyzer (Agilent), quantified with a Qubit fluorimeter (Invitrogen), and spiked in with 10% of φX 174 (φX) bacteriophage DNA library to balance for AT/GC content. Libraries were loaded on a rapid-mode flow cell and sequenced inside a HiSeq 2500 machine (Illumina), which produced ∼100 M single reads of 100 nt each per experiment (SR100) (Supplemental Table S3). The quality of raw FastQ files was assessed by FastQC (version 0.11.2).

### Bioinformatics analysis

The output of the sequencing machine was a large text file that contains the reads of a 100-nt barcoded luciferase fragment coming from all of the pooled experimental samples. To identify the barcodes that were induced by the drugs and not in the controls, the following steps were implemented. The reads from different samples were separated (demultiplexing). For each sample, the barcode region was isolated, and the number of barcodes was counted. Finally, the barcode counts of different experiments were joined in a single table, normalized on the median, and expressed as fold changes with regard to the average read counts of controls. Fold changes were ranked according to the average of the time series (sum of reads at 1, 2, 4, 8 h divided by 4 for both drug- and DMSO-treated samples) to identify barcodes that were more efficiently induced by the drugs than by DMSO (control).

#### Barcode counts

FastQ files were uploaded to the Galaxy server (Goecks et al. 2010) and converted to FastA. The luciferase 3′ UTR was clipped away starting from the XbaI site, isolating the 3′ end of the barcode. Clipped FastA files were demultiplexed using the barcode splitter tool: On the 5′, the identifier sequence (index) was used to assign every read to the corresponding experimental sample. Next, demultiplexed FastA files were trimmed from position 27 to 46 to isolate the barcode region. Finally, for each sample, trimmed barcodes were counted using FastA collapse, converted to a tabular file, and joined together in a table containing the barcode sequence in the first column and the barcode counts for each sample in the other columns. To filter out spurious sequences originating from sequencing errors, we kept only the 3065 barcode sequences present in all experiments and for which the promoter association was known (Supplemental Table S1).

#### Fold changes

The read count table was downloaded from Galaxy and imported into R Studio. An R script is available on GitHub (https://github.com/randogp/STARprom). We normalized the reads by dividing each barcode count with the median of the sample (column-wise) and then multiplying it by the median of all of the reads of the experiment. Fold changes were calculated by dividing normalized read counts by the average of the controls and were ranked according to the ratio between the average of “drug reads” and “control reads.” Fold changes are in Supplemental Table S4. Plasmids with barcodes that were induced in both biological replicates were selected for individual tests.

### Promoter retrieval

Two BC-STAR-PROM sublibraries were linearized by independent digestion with ApaI, FspI, or HincII. Digestions were heat-inactivated for 15 min at 70°C and pooled. A reverse primer matching the barcode region was annealed to each library in a 25-µL reaction containing 2 pmol of 5′-biotinylated reverse primer, 50 ng of linear DNA, 0.4 mM dNTPs, 5% DMSO, 2 U of Taq polymerase, and 1× Taq buffer (50 mM Hepes/KOH at pH 7.9, 50 mM KCl, 1.5 mM MgCl_2_). The reaction was incubated for 5 min at 95°C (denaturation) and 10 min at 60°C (annealing and extension). Extended primers were coupled to streptavidin beads in 400 µL of buffer A (Tris-HCl 10 mM, EDTA 1 mM, NaCl 0.5 M at pH 8.0) containing 20 µL of Dynabeads M-280 Streptavidin (Invitrogen). The coupling reaction was incubated at for 2 h room temperature on a rotating wheel (40 rpm), and the beads were immobilized on a magnetic stand. To remove uncoupled DNA, the beads were washed three times for 10 min in 500 µL of buffer A + 0.5% SDS, three times in buffer A, and once in Taq buffer. Beads were resuspended in 25 µL of Taq buffer, and the captured DNA was amplified with the primer 5′-TCTACTAGTCATCTTTTTGATGG-3′ and the appropriate reverse barcode primer. Amplified DNAs were digested with HindIII and NheI, and the promoter fragments were gel-purified and cloned into the destabilized luciferase vector pGL4.24 (Promega). Clones were verified by Sanger sequencing (Fasteris).

### Bioluminescence time-lapse microscopy

Cells were plated in µ-Dishes 35 mm high, ibiTreat (81156 from Ibidi) at a low density (100,000 cells unless otherwise stated) in 2 mL of 20% DMEM, HEPES (without phenol red) (GIBCO), and 200 µM luciferin. The dish was placed in a 37°C chamber equilibrated with humidified air containing 5% CO_2_, and time-lapse bioluminescence microscopy was performed using an Olympus LV200 workstation (Bioimaging Platform) equipped with a 20× objective. For each movie, bioluminescence emission was detected for several consecutive days using an EM-CCD camera (Image EM X2 9100-23B, Hamamatsu), taking images every 15 min and using exposure times of 10 min. The image series were analyzed using ImageJ software (Livak and Schmittgen 2001; Schneider et al. 2012). Bioluminescence images (512 × 512 tif stacks of 16 bits) were filtered from noise by taking for each pixel the minimum value between two consecutive acquisitions. For tracing experiments, bright-field images (512 × 512 pixels, 8-bit stacks) were corrected for uneven illumination by subtracting the background estimated with a median matrix of 8 pixels. A maximum matrix of 1 pixel was subsequently applied to facilitate cell tracking. Cells were tracked using the semiautomated CGE plug-in (Sage et al. 2010). The traces between two cell divisions, as manifested by cytokinesis, were imported on the bioluminescence images, and the luciferase signals were measured as intensity counts. For counting experiments, luminescence files were treated using the same parameters (contrast/brightness/threshold), and the tool “analyze particles” of ImageJ software was used to count luminescent cells for each frame of the movie.

### Data access

Raw FastQ and FastA files were deposited in NCBI's Gene Expression Omnibus (GEO) (Edgar et al. 2002) and are accessible through GEO series accession number GSE81271.

## Supplementary Material

Supplemental Material
