# Supplementary material for: Unbiased identification of signal-activated transcription factors by barcoded synthetic tandem repeat promoter screening (BC-STAR-PROM)
Source: Genes Dev. 2016 Aug 15;30(16):1895–907. doi: 10.1101/gad.284828.116 (PMC5024686; doi:10.1101/gad.284828.116)
Supplement: Supplemental Material [file supp_30_16_1895__index.html]

Supplemental Material 

# Unbiased identification of signal-activated transcription factors by barcoded synthetic tandem repeat promoter screening (BC-STAR-PROM)

## Supplemental Material

- Supplemental\_data.pdf
- Supplemental\_Table\_S1.xlsx
- Supplemental\_Table\_S6.xls
- Supplemental\_Table\_S4.xls
- Supplemental\_Table\_S5.xlsx
- Supplemental\_Movie1.avi
- Supplemental\_Movie4.avi
- Supplemental\_Movie2.avi
- Supplemental\_Movie5.avi
- Supplemental\_Movie3.avi
- Supplemental\_Movie6.avi
