# Supplementary material for: Unbiased identification of signal-activated transcription factors by barcoded synthetic tandem repeat promoter screening (BC-STAR-PROM)
Source: Genes Dev. 2016 Aug 15;30(16):1895–907. doi: 10.1101/gad.284828.116 (PMC5024686; doi:10.1101/gad.284828.116)
Supplement: Supplemental Material [file supp_30.16.1895_Supplemental_data.pdf]

Unbiased identification of signal-induced transcription factors by Barcorded Synthetic TAndem Repeat Promoter screening (BC-STAR-PROM)

Pauline Gosselin, Gianpaolo Rando, Fabienne Fleury-Olela, Ueli Schibler

A

(1) $$F_{0-n} = \frac{2}{4^N} \sum_{i=0}^{n(\leq N)} \frac{N!\,3^i}{i!(N-i)!}$$

(2) $$p = 1-(1-F_{0-n})^Z$$

B

For Z= 200,000 bp

N	*p*		
	n 0	n 0-1	n 0-2
7	>0,999	>0,999	>0,999
8	>0,999	>0,999	>0,999
9	0,783	>0,999	>0,999
10	0,317	>0,999	>0,999
11	0,091	0,961	>0,999
12	0,024	0,586	>0,999
13	0,006	0,212	0,988
14	0,001	0,062	0,723

Supplemental Figure S1: Estimation of the probability to find TF binding sites in random DNA

(a) (1) Equation (Pascal's binominal theorem) used to calculate the statistical frequency (F0-n) of a non-palindromic TF binding site spanning N bp and containing between 0 and n mismatches in random double-stranded DNA (i describes a series of n values). (2) Equation used to calculate the probability (p) of finding at least one such TF binding site in Z bps of double stranded random DNA.

(b) Table presenting probabilities (p) of finding TF binding sites spanning from 7 to 14 bp (N) and allowing for n mismatches in Z = 200,000 bp of double stranded random DNA (which corresponds to a total of 400,000 bp, if both strands are counted).

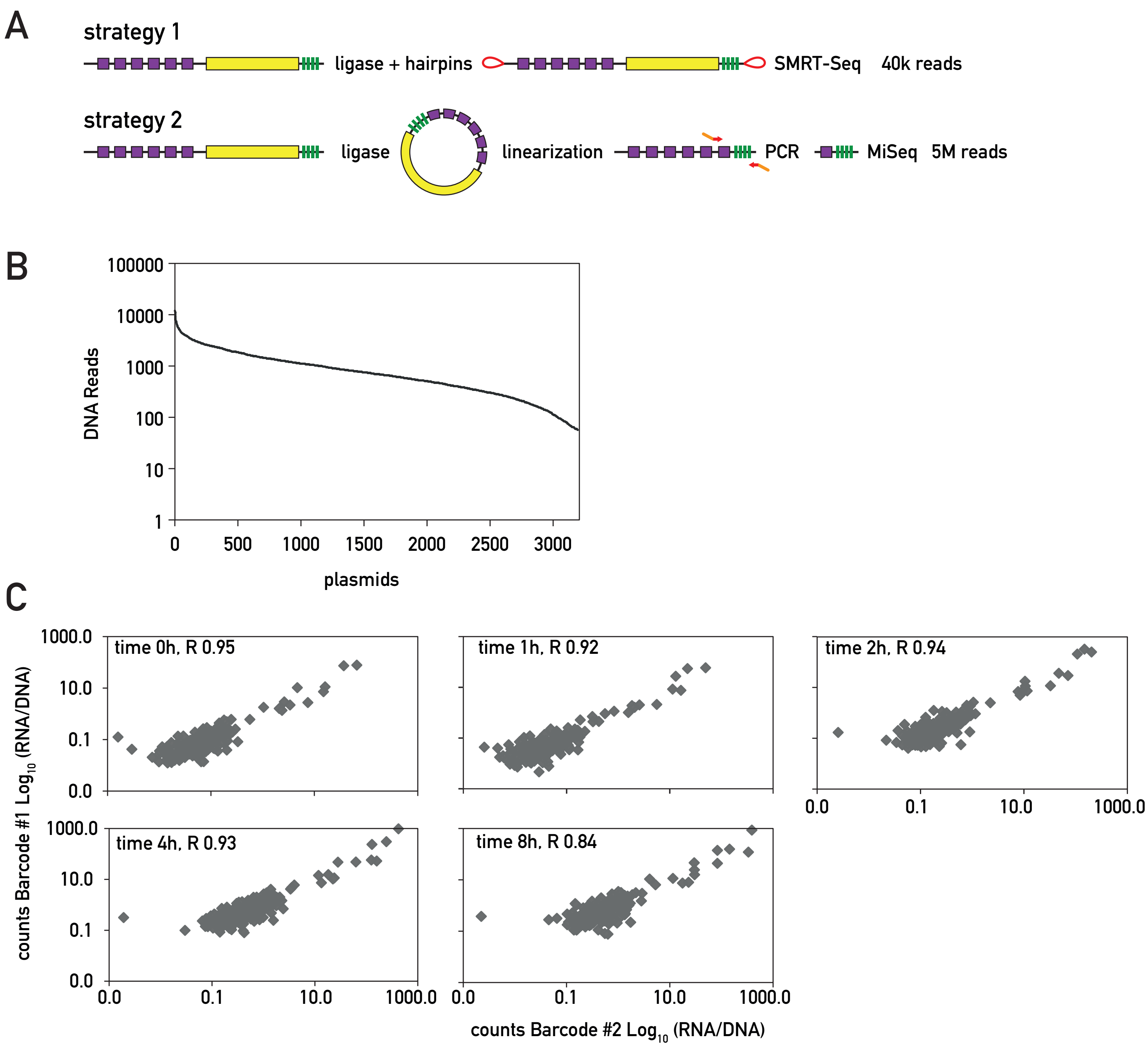

Supplemental Figure S2: Determination of the promoter-barcode association

(A) Promoter-barcode association strategy. Two experimental sequencing strategies were employed to determine barcodes-promoter associations. Strategy 1: SMRT hairpin adaptors (Pacific Biosciences) were ligated to both ends of the NheI-XbaI restriction fragments of 2.3kb encompassing all six promoter repeats, the luciferase reporters, and the barcodes. These fragments were sequenced by the PacBio SMRT method. Strategy 2: Linear promoter-luciferase-barcode restriction fragments (NheI, XbaI) were circularized by intermolecular ligation, and the barcode-promoter junctions were amplified by PCR. To associate each barcode to the corresponding promoter, a 150 bp region spanning the barcode and the first promoter repeat was sequenced on an Illumina MiSeq lane (5M reads).

(B) Read count distribution for the 3363 barcodes whose promoter associations have been confirmed by both strategies. We counted 3,237 barcodes that were associated with 2,894 promoters with at least 50 reads coverage.

(C) BC-STAR-PROM quality control: For each promoter associated with 2 different barcodes (Supplemental Table S2), we plotted the expressed reads of one barcode against the expressed reads of the other barcode. The RNA reads have been divided by the DNA reads measured in the plasmid transfection mix. The scatterplots suggest high reproducibility (Pearson > 0.9).

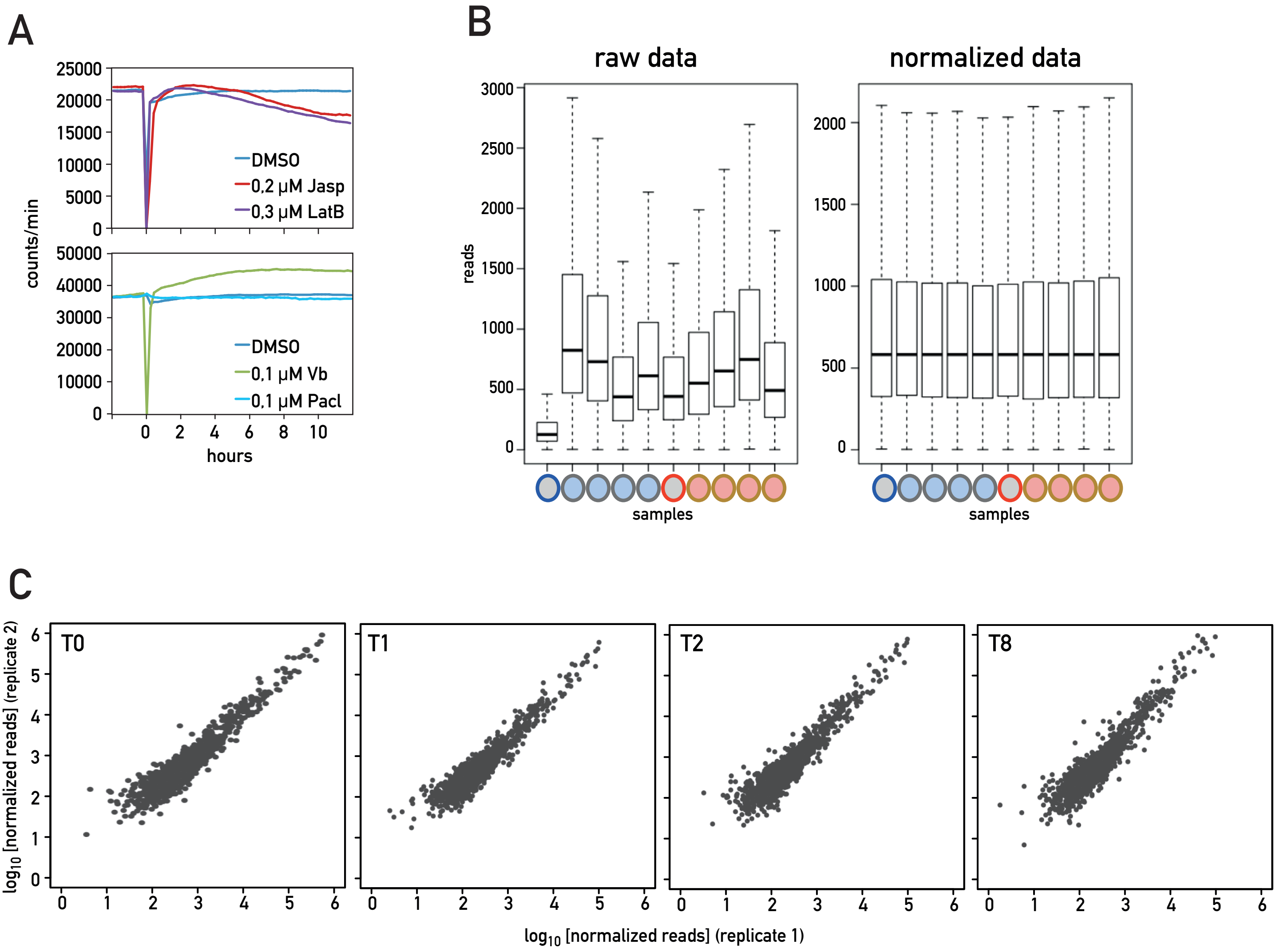

Supplemental Figure S3: BC-STAR-PROM analysis

(A) BC-STAR-PROM screening. The entire BC-STAR-PROM library was transfected into U2OS cells, and the response to cytoskeletal perturbations was tested by real-time luciferase recordings. Total luminescence counts per minute are plotted against time after drug induction. As no significant induction can be observed, the minority of induced plasmids did not contribute to a number of photons sufficient to affect the bioluminescence profiles of the entire library after drug treatments (Jasplakinolide (Jasp), LatrunculinB (LatB), Vinblastine (Vb), Paclitaxel (Pacl)).

(B) BC-STAR-PROM read distribution. For each sample barcode counts were divided by the sample median and multiplied by the median of all the counts of the experiment, thereby normalizing for pooling and amplification biases. This normalization assumes that the median expression level of the whole library does not change during the experiment, as indicated by the experiments displayed in panel **(A)**. On the boxplot, the black line is the median, the bottom and top of the box are the 25th and 75th percentiles of the distribution, respectively, and the whiskers delimit the maximum and minimum values.

(C) BC-STAR-PROM reproducibility. Barcode read counts are plotted for two biological replicates at different time points of DMSO treatment. The Pearson correlation coefficients are: 0.9319 (time 0); 0.9275 (time 1), 0.9188 (time 2), 0.9195 (time 8).

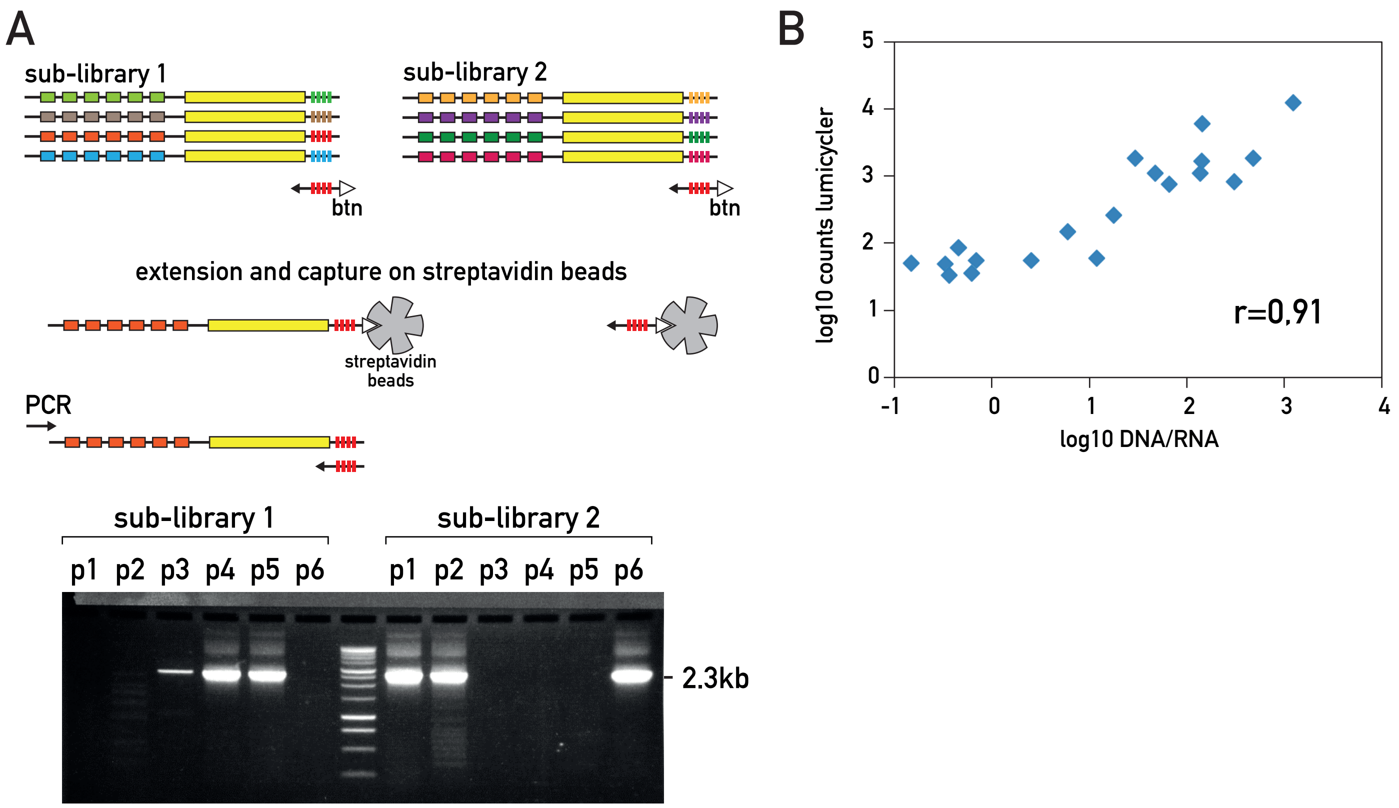

Supplemental Figure S4: Promoter retrieval

(A) Promoter retrieval strategy: BC-STAR-PROM clones of interest were isolated from either of the two linearized and denatured library pools (see Material & Methods) by affinity purification and PCR amplification. A biotinylated primer with a sequence complementary to the corresponding barcode was annealed to the sub-library DNA containing the clone of interest and extended using Taq polymerase. Biotinylated primers were tested on both sub-libraries to identify the one containing the relevant plasmid. After coupling to streptavidin beads and extensive washing, the DNA region encompassing the barcode and the corresponding promoter repeats was PCR amplified. The promoter repeats were retrieved by restriction digestion and gel-purification, and cloned upstream of a basal promoter driving transcription of a destabilized luciferase. The sequences of the isolated promoter elements were verified by Sanger sequencing. As shown by agarose gel electrophoresis of amplified DNA, a given promoter-barcode combination was only present in one of the two sub-libraries, as expected.

(B) Correlation of the RNA barcode reads [corrected for the abundance of the corresponding expression vectors in the library (Supplemental Table S1)] and bioluminescence counts for 19 isolated promoters. The values represent averages determined in six experiments before drug treatments. The Pearson correlation coefficient is 0.91.

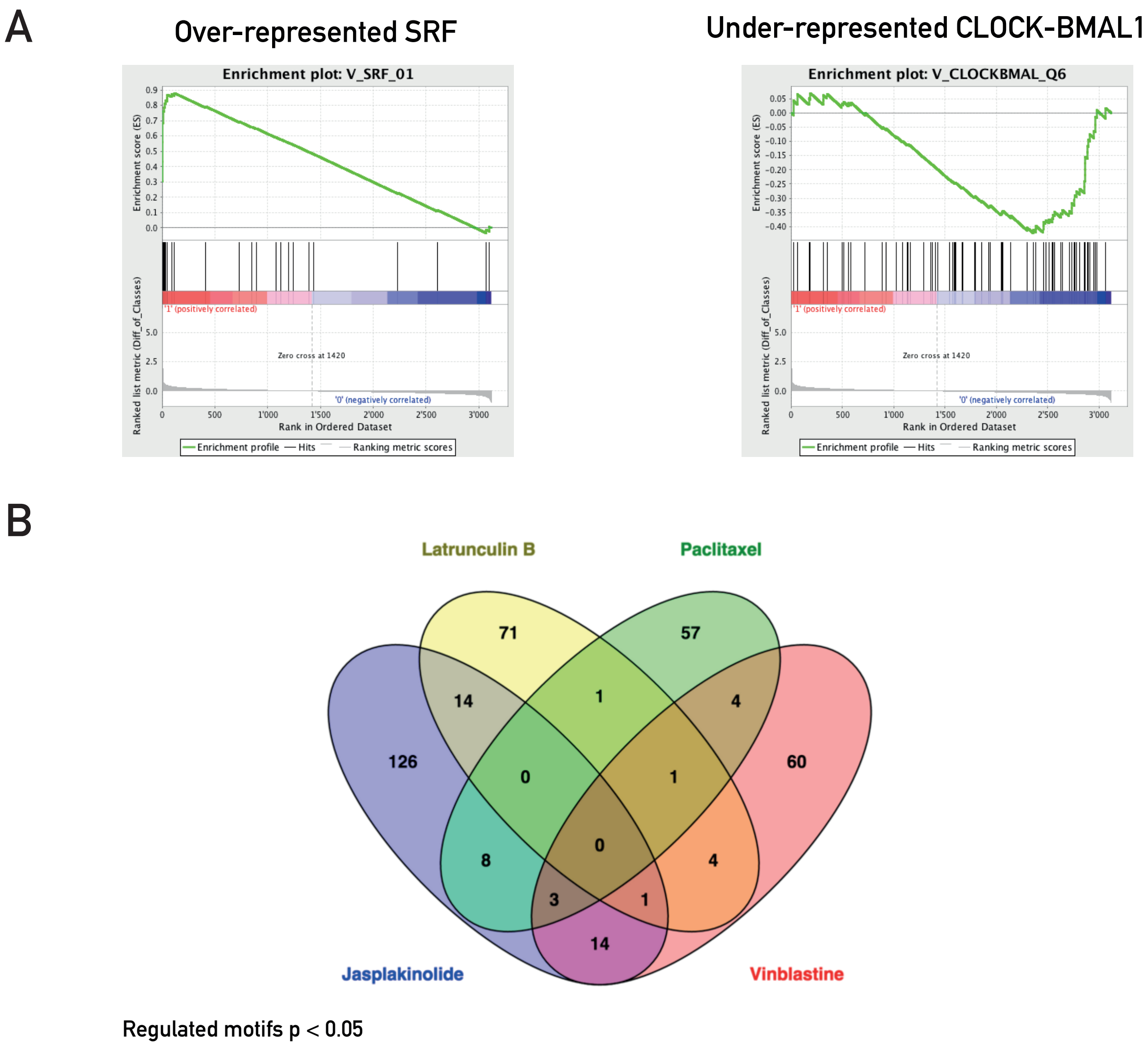

Supplemental Figure S5: Motifs enrichment analysis

(A) Graphs showing the enrichment plot generated during our motifs enrichment analysis for SRF and CLOCK-BMAL1 motifs. Analysis was performed on ranked promoter expressions that are up-regulated (red) or down regulated (blue) during Jasplakinolide treatment.

(B) Venn diagram showing the common motifs whose activities are regulated by the different drugs.

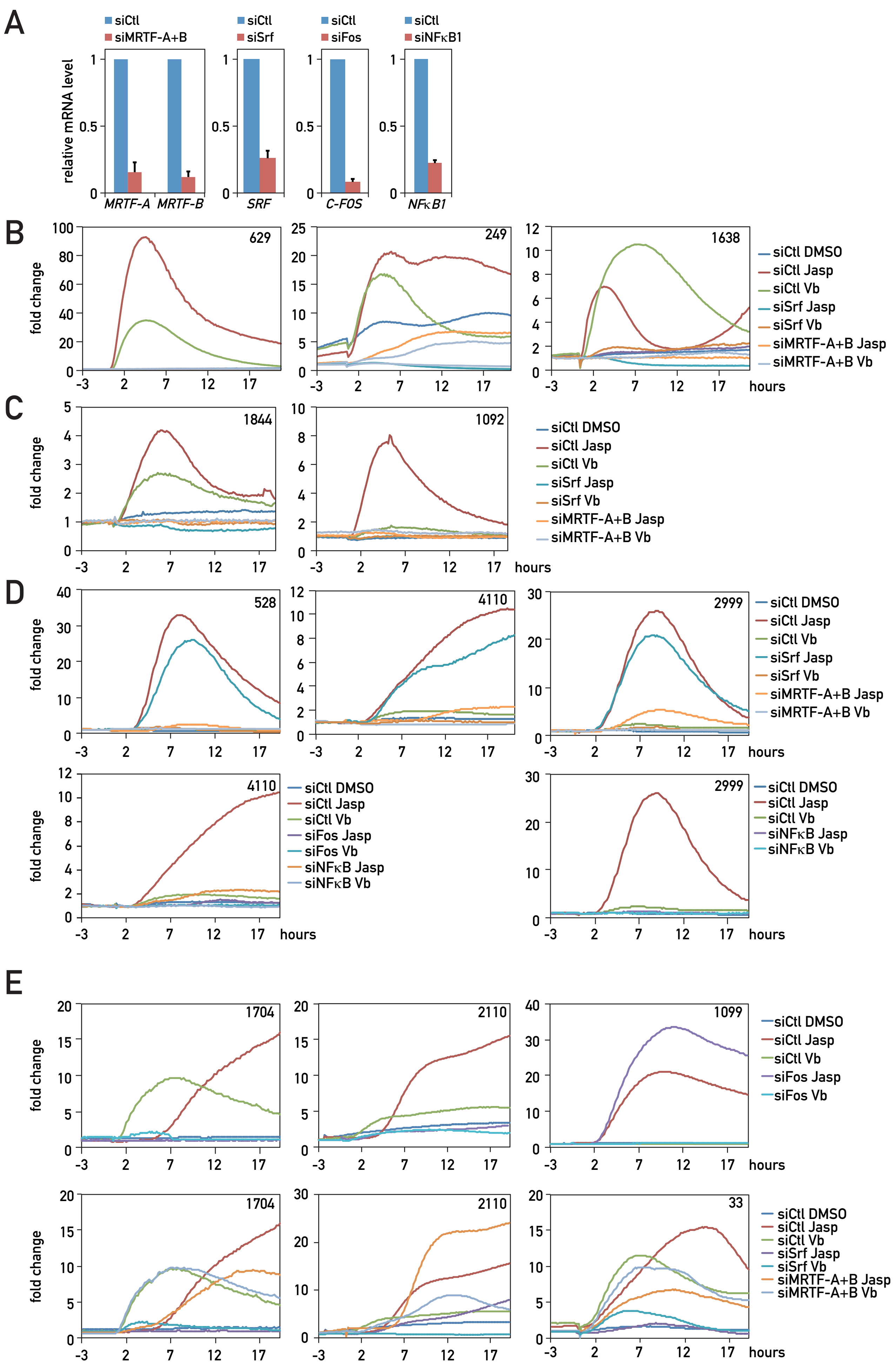
A
siCtl
siMRTF-A+B
siCtl
siSrf
siCtl
siFos
siCtl
siNFκB1
relative mRNA level
MRTF-A
MRTF-B
SRF
C-FOS
NFκB1
B
fold change
629
249
1638
siCtl DMSO
siCtl Jasp
siCtl Vb
siSrf Jasp
siSrf Vb
siMRTF-A+B Jasp
siMRTF-A+B Vb
hours
C
1844
1092
D
528
4110
2999
siFos Jasp
siFos Vb
siNFκB Jasp
siNFκB Vb
E
1704
2110
1099
33

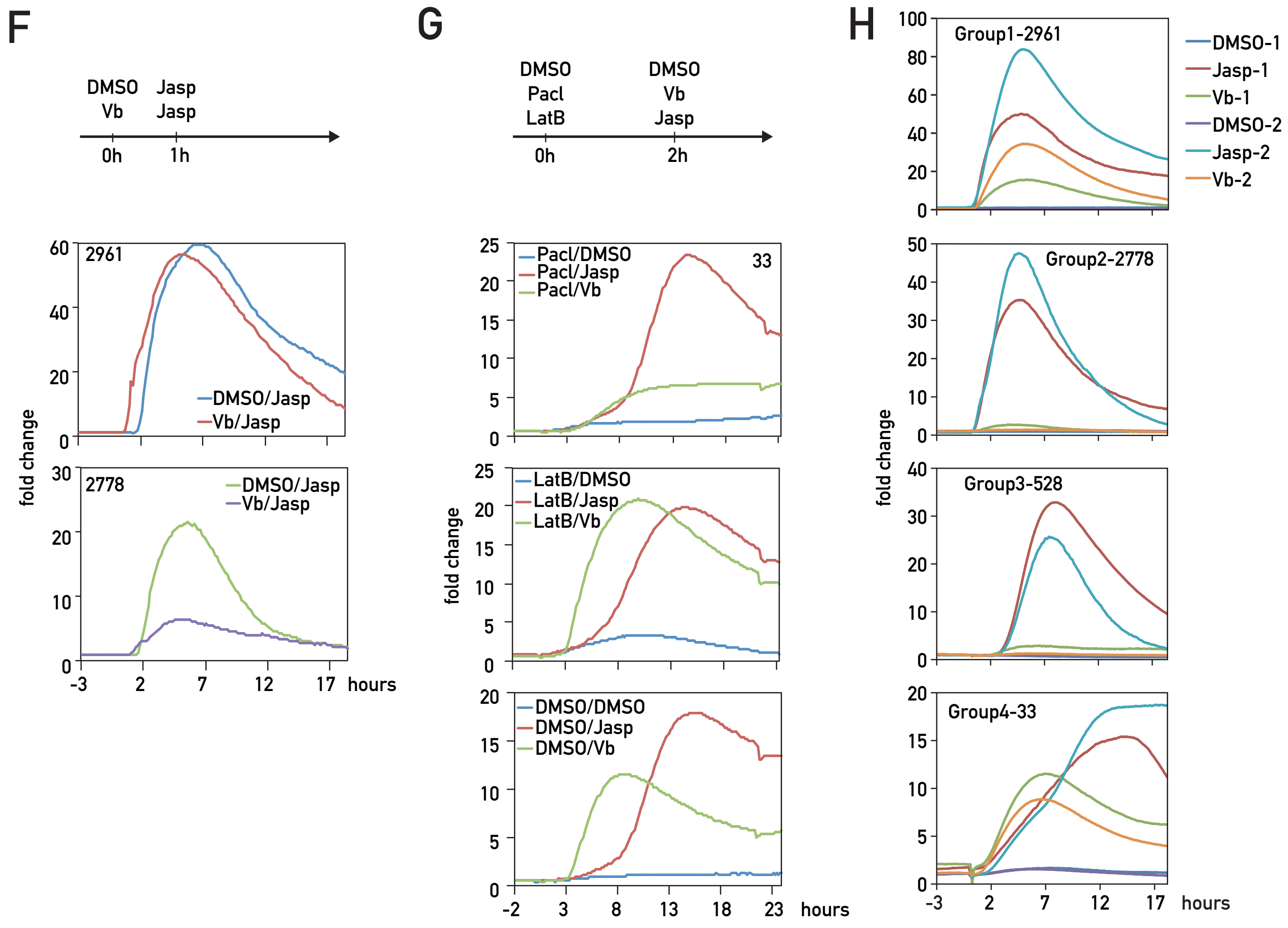

Supplemental Figure S6: Validation of transcription factor identifications

(A) The knockdown efficiency of each siRNA was obtained by qPCR (mean mRNA levels +/- SD) from three independent transfections with non-targeting siRNAs (siCtl) or specific siRNAs (siMRTF-A+B, siSrf, siNFkB1, siFos), normalized to *GAPDH* mRNA.

Individual STAR-PROM clones were co-transfected into U2OS cells with siRNAs targeting the indicated TFs, and the responses to cytoskeletal perturbations were assessed by real-time luciferase recordings for promoters of group-1 (**B**), group-2 (**C**), group-3 (**D**) and group-4 (**E**).

(F) Vinblastine prevents SRF induction by Jasplakinolide. U2OS cells, transfected with plasmid 2778 (or with 2961 as a control), were pretreated with Vinblastine or DMSO one hour before Jasplakinolide treatment, and luminescence recordings are plotted against time (after the pretreatment).

(G) Jasplakinolide and Vinblastine activate group-4 promoters via independent pathways. U2OS cells transfected with plasmid 33 were pretreated with DMSO, 1 M Paclitaxel, or 0,3 M LatB in order stabilize microtubules or destabilize actin networks, respectively, two hours before Vinblastine or Jasplakinolide treatment. Luminescence recordings are plotted against time (after pretreatment).

(H) Two experimental replicates are shown for the representative promoters of each group.

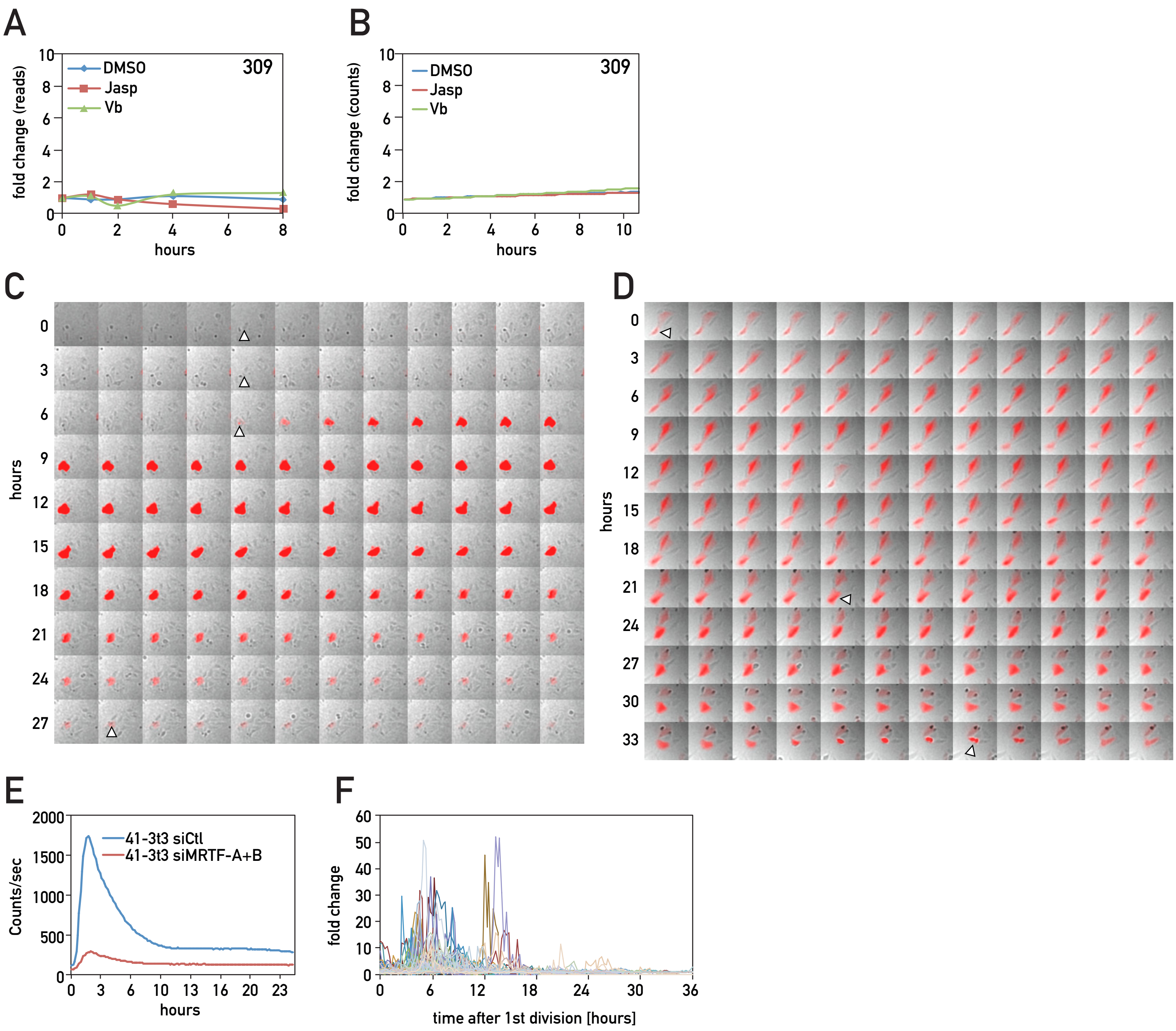

Supplemental Figure S7: Endogenous signals activate SRF during cell cycle

(A) Fold changes of RNA barcode reads obtained for plasmid 309 in BC-STAR-PROM experiment.

(B) U2OS cells were transfected with plasmid 309, starved for 24h and induced with Jasplakinolide or Vinblastine. Fold changes of photon counts are plotted against time after drug treatment.

(C) and **(D)** Representative luminescence recordings (luminescence in red) during time lapse microscopy of one U2OS cell transfected either with plasmid 296 (**C**) (movie 1) (responding to Jasplakinolide and Vinblastine) or with plasmid 309 (**D**) (movie 2) between two cell divisions. The recorded cell is indicated by white arrow heads.

(E) 41-3t3 cells transfected or not with siRNA against MRTF-A+B were seeded at near confluence in DMEM+ 20%FBS, and luminescence was recorded in a lumicycler in order to determine the timespan of serum induction (SRF responds to serum stimulation).

F) The fold changes of luminescence counts for each of the 100 traces shown in **Figure 6E** were calculated by dividing each count by the median of the entire trace recorded for an entire cell division cycle (cytokinesis to cytokinesis).

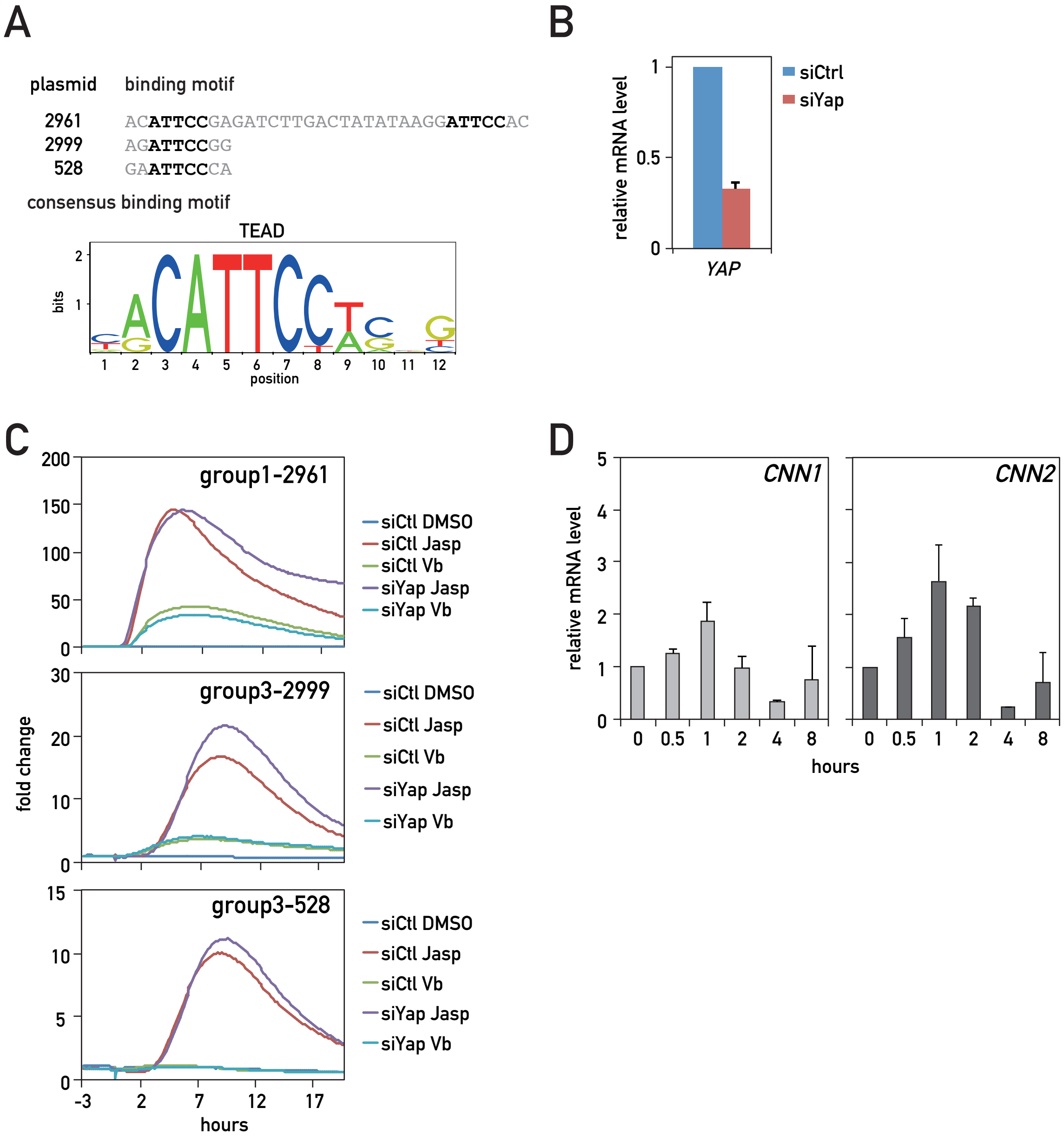

Supplemental Figure S8. TEAD motifs in BC-STAR-PROM plasmids do not mediate a strong induction by Jasplakinolide

(A) TEAD motifs found with Jaspar in the top 20-induced promoters sequences.

(B) Knockdown efficiency of Yap siRNA obtained by qPCR calculated in three independent transfections experiments.

(C) Induced BC-STAR-PROM vectors containing TEAD motifs were co-transfected into U2OS cells with Yap siRNA or Control siRNAs (Ctl), and their responses to cytoskeletal perturbations were examined by real-time luciferase recordings.

(D) Transcripts encoded by the YAP target genes *CNN1* and *CNN2* were quantified by RT-qPCR in RNA samples collected during the time-course of replicate 1 after Jasplakinolide treatment. SDs are from three technical replicates (i.e. three RT-qPCR determinations from the same RNA samples).

Promoters with 1 barcodes	2364 promoters
Promoters with 2 barcodes	235 promoters
Promoters with 3 barcodes	28 promoters
Promoters with 4 barcodes	4 promoters
Promoters with 5 barcodes	0 promoters
Barcodes matching 0 promoters	13 barcodes
Barcodes matching ≥ 2 promoters	0 barcodes

Supplemental Table S2: Number of promoters associated with different barcodes and vice versa, in the 2631 promoters identified in the MiSeq data (displaying more than 100 reads).

Library	drug	Illumina	Mode	raw reads
lib4	Jasplakinolide	HiSeq	sr100	123,444,418
lib5	Latrunculin B	HiSeq	sr100	115,022,531
lib7	Paclitaxel	HiSeq	sr100	35,203,769
lib3	Vinblastine	HiSeq	sr100	102,881,362
lib9	Biological duplicates for Jasplakinolide and Vinblastine	HiSeq	sr100	135,603,719

Supplemental Table S3: Next Generation Sequencing experiments

MOVIES LEGENDS

Movie 1. Plasmid 2961 is expressed only during a restricted period during two cell divisions.

U2OS cells, transfected 24hours earlier with plasmid 2961, were seeded at a low density (100,000 cells) in white DMEM 20% FBS. In order to eliminate confounding effects generated by the serum induction triggered by exposing the cells to new medium, monitoring was started 16 hours after seeding. Individual cells were recorded by real-time bioluminescence imaging during 54 hours, using the Olympus LV200 microscope and a 20X objective. Images were taken every 15 minutes (one frame), and the movie is displayed at 7 frames/second. Bioluminescence is shown in red.

Movie 2. Plasmid 309 displays constant expression during cell cycle in U2OS diving cells.

U2OS cells, transfected 24 hours earlier with plasmid 309, were seeded at a low density (100,000 cells) in white DMEM 20% FBS. Individual cells were recorded by real-time bioluminescence imaging during 54 hours using the same settings as for movie 1.

Movie 3. 41-3t3 cells display a recurrent induction of the SRF-luc reporter gene after cell division

41-3t3 were seeded at low density (100,000 cells) in white DMEM 20% FBS. Individual cells were directly recorded one hour after seeding by real-time bioluminescence imaging for 75 hours using the ultrasensitive Olympus LV200 microscope and a 20X objective.

Movie 4. SRF-Luc induction ceases when 41-3t3 cells reach confluency.

41-3t3 cells were seeded at higher density than that used in movie 3 (400,000 cells) in white DMEM 20% FBS and recorded by real-time bioluminescence imaging for 45 hours (using the same settings as for movie 3).

Movie 5. 41-3t3 transfected with siCtl

41-3t3 cells, transfected 48 hours earlier with a non-targeted siRNA (siCtl), were seeded at low density (100,000 cells) in white DMEM 20% FBS. Individual cells were recorded by real-time bioluminescence imaging for 78 hours (using the same settings as for movie 3).

Movie 6. The induction of SRF-luc reporter gene during cell division dampens in 41-3t3 cells transfected with siRNA against MRTF

41-3t3 cells, transfected 48 hours earlier with siRNA against MRTF-A+MRTF-B, were seeded at low density (100,000 cells) in white DMEM 20% FBS. Individual cells were recorded by real-time bioluminescence imaging for 78 hours (using the same settings as for movie 3).

Supplemental Material

Motifs enrichment analysis

In our adaptation of the Gene Set Enrichment Analysis (GSEA) framework developed by Subramanian and colleagues (Subramanian et al. 2005), the “genes” are replaced by the promoters’ sequences and the “sets” are formed by grouping all the promoters containing a given motif. We built 2110 sets, using FIMO (Grant et al. 2011) to identify 105,791 occurrences for binding motif ($p < 10^{-4}$, that covered 93% of the 2110 motifs described in the Transfac 2012 database). Then, we determined which motifs were primarily enriched at the top or the bottom of the drug response rank, performing an enrichment analysis essentially as described (Subramanian et al. 2005): i) we ranked all promoter’s fold changes on a list that measures the promoter’s correlation with a given drug treatment, ii) we identified the rank positions of all promoters of any promoter set, and iii) for each set, we calculated an enrichment score that reflects the degree to which a given set is overrepresented at the extremes of the ranked list. Supplemental Fig. 8A shows as example the distribution of one motif over-represented (SRF) and one motif under-represented (CLOCK-BMAL1) after Jasplakinolide treatment.

To establish the significance, and exclude promoter sets that could be enriched by chance, we randomized the drug/dmso sample labels and retested for enrichment 1,000 times. Finally, we adjusted for multiple hypothesis testing using the standard GSEA false discovery rate calculation. The promoter sets that significantly outperformed iterative random class permutations were considered significant.

When perturbing actin dynamics, we identified 243 motifs ($p < 0.05$): 166 motifs responded to Jasplakinolide (actin stabilization) and 92 motifs responded to Latrunculin B (actin destabilization). Perturbing the microtubules influenced 153 motifs ($p < 0.05$): 74 responded to Paclitaxel (microtubule stabilization) and 87 responded to vinblastine (microtubule destabilization). The Venn diagram (Supp. Fig 8B) shows that in general, different drugs regulated different motifs. Regulated motifs are given in Supplemental Table S6. »

Grant CE, Bailey TL, Noble WS. 2011. FIMO: scanning for occurrences of a given motif. *Bioinformatics* **27**: 1017-1018.

Subramanian A, Tamayo P, Mootha VK, Mukherjee S, Ebert BL, Gillette MA, Paulovich A, Pomeroy SL, Golub TR, Lander ES et al. 2005. Gene set enrichment analysis: a knowledge-based approach for interpreting genome-wide expression profiles. *Proc Natl Acad Sci U S A* **102**: 15545-15550.
